# The AT-hook motif-encoding gene *METABOLIC NETWORK MODULATOR 1* underlies natural variation in *Arabidopsis* primary metabolism

**DOI:** 10.3389/fpls.2014.00415

**Published:** 2014-08-22

**Authors:** Baohua Li, Daniel J. Kliebenstein

**Affiliations:** Department of Plant Sciences, University of CaliforniaDavis, CA, USA

**Keywords:** primary metabolism, QTL, TCA, AT-hook motif, Arabidopsis

## Abstract

Regulation of primary metabolism is a central mechanism by which plants coordinate their various responses to biotic and abiotic challenge. To identify genes responsible for natural variation in primary metabolism, we focused on cloning a locus from *Arabidopsis thaliana* that influences the level of TCA cycle metabolites *in planta*. We found that the Met.V.67 locus was controlled by natural variation in *METABOLIC NETWORK MODULATOR 1* (*MNM1*), which encoded an AT-hook motif-containing protein that was unique to the Brassicales lineage. *MNM1* had wide ranging effects on plant metabolism and displayed a tissue expression pattern that was suggestive of a function in sink tissues. Natural variation within *MNM1* had differential effects during a diurnal time course, and this temporal dependency was supported by analysis of T-DNA insertion and over-expression lines for *MNM1*. Thus, the cloning of a natural variation locus specifically associated with primary metabolism allowed us to identify *MNM1* as a lineage-specific modulator of primary metabolism, suggesting that the regulation of primary metabolism can change during evolution.

## Introduction

An organism's growth and fitness within an environment is largely determined by its ability to efficiently obtain and utilize energy and nutrients. Central to this process is primary metabolism, which determines the use of inputs from the environment to produce all of the necessary building blocks for cells and the resulting biomass. To optimize fitness, primary metabolism must be precisely tuned and coordinated to make the most efficient use of available resources. This basic supposition is central to a wide range of biological fields, from the study of organismal growth to the study of organismal/environment interactions (Karban and Baldwin, 1997; Smith and Stitt, 2007). The best understood regulatory mechanism for primary metabolism involves allosteric control, in which substrate availability and product levels directly influence enzyme activities. Allosteric mechanisms allow rapid optimization of metabolic fluxes, and, theoretically, optimize energy utilization for biomass production; however, this regulatory mechanism is largely limited to internal rebalancing of primary metabolism.

Recent work has revealed other key regulatory mechanisms coordinating primary metabolism and growth. These include regulatory links whereby the circadian clock controls key components of central metabolism and coordinates daily growth with energy availability (Nozue et al., 2007; Covington et al., 2008; Gutierez et al., 2008; Fukushima et al., 2009; Harmer, 2009; Graf et al., 2010; Pracharoenwattana et al., 2010). This coordination occurs via direct molecular links between the circadian clock and primary metabolism. In addition, transcriptional analysis of circadian-responsive transcripts shows that primary metabolism likely has more regulatory inputs than simply the circadian clock (Harmer et al., 2000; Harmer and Kay, 2005).

Regulation of central metabolic pathways like the TCA cycle appears to be distributed across a number of enzymes providing distinct and independent regulatory inputs (Araujo et al., 2012). The existence of numerous regulatory inputs into a single pathway may provide the organism with a highly intricate system to modulate the flux into and out of central metabolism. Accumulating evidence indicates that organisms have developed numerous mechanisms enabling primary metabolites to self-regulate, including via transcriptional regulation of their own biosynthetic pathways (Larkin et al., 2003; Xiao et al., 2012; Finkemeier et al., 2013). Given the central importance of primary metabolism to an organism, it is likely that these initial observations on the regulation of and by primary metabolism are only a hint at the vast underlying regulatory networks. Thus, there are likely a large number of unidentified regulatory components that can influence primary metabolism.

In the past decade, the study of natural variation has become a key tool to elucidate metabolic networks and their regulation (Keurentjes et al., 2006, 2008; Schauer et al., 2006, 2008; Sulpice et al., 2009; Chan et al., 2010; Matsuda et al., 2012; Riedelsheimer et al., 2012; Li et al., 2013). Most natural alleles affecting primary metabolism are quantitative in nature and not lethal, whereas most induced mutants are either lethal or produce dwarfed plants. Thus, natural variation provides the ability to genetically perturb primary metabolism and identify the consequences without killing the plant. Analysis using natural variation has suggested a direct link between metabolomic variation and the growth of *Arabidopsis thaliana* accessions under a variety of conditions (Sulpice et al., 2009). Naturally variable loci that mediate differences in primary metabolism form epistatic networks in larger populations and their effects on primary metabolism depend upon the time of day (Rowe et al., 2008; Chan et al., 2010). Thus, identifying the genes underlying natural variation in primary metabolism may uncover components of the regulatory network influencing primary metabolism.

Analysis of metabolomic variation in the Bay × Sha recombinant inbred line (RIL) population of *Arabidopsis thaliana* identified a number of QTLs for natural variation in the plant metabolome (Rowe et al., 2008). Previous efforts identified *ELF3* and *AOP2* as key genes controlling natural variation underlying QTLs for both primary and secondary metabolism (Jimenez-Gomez et al., 2011a,b; Kerwin et al., 2011). These QTLs were not specific to the TCA cycle, whereas variation at the Met.V.67 locus was causing significant alteration in steady-state levels of the primary metabolite succinate (Rowe et al., 2008). Transcriptomic analysis of the same RIL population harvested at the same time of day showed that there was strong concordance in the location of QTLs controlling the expression of the circadian clock output networks with these metabolomic QTLs (West et al., 2007; Kerwin et al., 2011). This concordance was due to variation at known circadian loci likely altering metabolic networks, i.e., *ELF3*, and known metabolic loci altering the circadian oscillator, i.e., *AOP* (Jimenez-Gomez et al., 2011a,b; Kerwin et al., 2011). We have previously shown that genes with cis-eQTLs (natural variation in gene expression that map to their physical position) often identify causal loci for QTLs (Kliebenstein, 2008, 2009). Thus, we theorized that we could identify candidate genes for the remaining unknown metabolomic loci by searching for genes that are affiliated with the circadian clock and contain a cis-eQTL.

In this work we report the identification and initial characterization of *METABOLIC NETWORK MODULATOR 1* (*MNM1*) encoding an AT-hook motif-containing protein that underlies the Met.V.67 QTL affecting the differential accumulation of TCA cycle intermediates within *Arabidopsis thaliana*. Analysis of T-DNA insertion lines and over-expression lines showed that they had opposite effects on flowering and growth, but highly similar effects on metabolism. Quantitative complementation from the Bay and Sha alleles in the Col-0 T-DNA insertion line backgrounds showed that the natural alleles in the AT-hook gene led to broad metabolic differences that focused on the TCA cycle with a time-of-day dependency in allelic effect. Translational fusions suggested that whereas *MNM1* had broad physiological and metabolomic effects, it was largely limited to sink tissues. Together, these data show that *MNM1* is causal for the Met.V.67 QTL and may act to coordinate central metabolism and physiology across the whole plant.

## Materials and methods

### Plant material and experimental conditions

For the metabolomics analysis of the T-DNA insertion lines and OE lines, Arabidopsis plants were grown in controlled-environment chamber at 20°C with 10 h light (noon to 10 p.m.) at 100–120 mE light intensity with two complete independent replicates of the whole experiment. The short-day conditions used were identical to those used to identify the Met.V.67 QTL allowing us to better compare results. Briefly, seeds were imbibed in water at 4°C for 3 days, and planted into Sunshine Mix 1 soil in a randomized complete block design. Seedlings were thinned to one plant per pot at 7 days after planting, for a total of 48 plants per block. At 6 weeks post germination, the chamber was set to continuous light conditions at relative dawn (actually noon), and the plant samples were harvested for metabolite analysis every 4 h across 2 days for T-DNA insertion lines and OE lines. In each experiment, four individual plants per genotype were harvested at each time point for sampling. This provides eight total samples per genotype per time point across the whole dataset.

For complementation analysis of the Bay and Sha alleles in the *mnm1-1* and *mnm1-2* backgrounds, homozygous T3 lines were used for each allele in each background. Plants were grown in controlled-environment chamber at 20°C with 10 h light (noon to 10 p.m.) at 100–120 mE light intensity with two complete independent replicates of the whole experiment. Briefly, seeds were imbibed in water at 4°C for 3 days, and planted into Sunshine Mix 1 soil in a randomized complete block design. Seedlings were thinned to one plant per pot at 7 days after planting. At 6 weeks after germination, the chamber was set at continuous light condition at relative dawn, and the plant samples were harvested for metabolite analysis at 4 h and 24 h post dawn. This provided 32 total samples per genotype per time point across the whole dataset.

### T-DNA lines and generation of transgenic plants

T-DNA lines of SALK_085140 and SALK_128695 were ordered from ABRC (Sussman et al., 2000; Alonso et al., 2003), and lines with homozygous insertions were identified by PCR-based genotyping (Table S1).

All constructs described below were confirmed by sequencing, and transgenic Arabidopsis plants were generated by the flower-dipping method using Agrobacterium GV3101 containing different constructs (Clough and Bent, 1998). All T1 plants were selected by Basta and allowed to self-pollinate to generate T2 lines. Individual T2 plants for each independent T1 were allowed to self-pollinate and tested for homozygosity in the T3 generation. T3 homozygous transgenic plants were confirmed by PCR-based genotyping and used for this study.

To generate the overexpression lines, the *MNM1* coding region was amplified from cDNA of Col-0 by primers of AKORF5 and AKORF3 (Table S1), cloned into pENTR/D-TOPO vector (Invitrogen), and subcloned to pB2GW7.0 destination vector by Gateway LR Clonase (Invitrogen). This leads to *MNM1* expression being driven by the 35S promoter. Lines showing dramatic morphological differences were kept as seeds but not used for molecular analysis.

To generate the complementation transgenes, the genomic regions of *MNM1* from Bay and Sha genomic DNA were amplified using the AKP5 and AK3Full primers (Table S1). These were then cloned into pENTR/D-TOPO vector, and subcloned into pMDC123 destination vector by Gateway LR Clonase. These constructs were transformed into both the *mnm1-1* and *mnm1-2* backgrounds to test for quantitative complementation.

To generate the GUS fusion lines, the promoters and coding regions of *MNM1* from Bay and Sha without stop codons were amplified by AKP5 and AKORF3A (Table S1), cloned into pENTR/D-TOPO vector, and subcloned to pBGWFS7 destination vector by Gateway LR Clonase (Table S1). All constructs were transformed into Col-0, *mnm1-1*, and *mnm1-2* backgrounds to allow for potential identification of differences in these backgrounds upon *MNM1* expression. The transgenic plants are confirmed by PCR-based genotyping.

### Flowering time

To measure flowering time in the lines with different *MNM1* genotypes, plants were arranged in a randomized complete block design within both short- and long-day conditions. For the long-day *mnm1-1* and *mnm1-2* comparison, the experiment was replicated five times with an average of 30 individuals per genotype per experiment such that there were 152 Col-0 individuals, 149 *mnm1-1* individuals and 157 *mnm1-2* individuals measured across the five experiments. For the short-day *mnm1-1* and *mnm1-2* plants, the experiment was replicated three independent times with a total of 49 Col-0 individuals, 48 *mnm1-1* individuals, and 47 *mnm1-2*individuals measured. The flowering of the *MNM1* OE genotypes was measured in long-day conditions with three replicated experiments providing measurements for a total of 54 Col-0 individuals, 52 OE7 individuals, and 54 OE8 individuals. Flowering time was recorded for all genotypes when the first flower opened. A secondary metric of flowering time, leaf number at flowering, was recorded only for the *mnm1-1* and *mnm1-2* lines because of the abnormal leaf phenotypes of overexpression lines.

### Histochemical GUS assay

Three independent transgenic lines containing either the Bay or Sha allele of the translational GUS fusion within the WT Col-0, *mnm1-1*, or *mnm1-2* backgrounds were stained every week for 6 weeks and pictures were taken each time. GUS staining of whole plants was done as previously described (Weigel and Glazebrook, 2002).

### RNA extraction, RT-PCR, and quantitative PCR

For qPCR and RT-PCR study, Arabidopsis plants were grown in a controlled-environment chamber at 20°C with 16 h light (6 a.m. to 10 p.m.), and 3-week-old plants were harvested at relative noon. One sample of each genotype was harvested and the entire experiment was replicated twice. All harvested samples were frozen in liquid nitrogen immediately and stored at −80°C until extraction. Total RNA was extracted using Trizol (Invitrogen), and 1 μg total RNA was treated with DNase I (Invitrogen). First strand cDNA was generated using the Reverse Transcription System (Promega) and used for RT-PCR and qPCR. Preliminary RT-PCR was performed using four pairs of primers for *MNM1* with *TUBULIN2* (AT5G62690) as a control (Table S1). Quantitative PCR was performed using the SYBR Green Kit in a 7300 Real Time PCR System (Applied Biosystems) with *UBQ10* (At4g05320), and *ACTIN2F* (At3g18780), as controls (Table S1).

### Metabolomics analysis

For each metabolomics sample, one leaf disk from each of two leaves per plant was harvested, providing two leaf disks of approximately 20 mg total weight. Unless otherwise noted, all harvesting started at subjective mid-day, finishing within 15 min. Each plant was independently harvested and extracted using previously published protocols (Weckwerth et al., 2004a,b; Meyer et al., 2007). The samples were stored dry at −80°C until automated derivatization and GC-TOF-MS analysis at the UC Davis Genome Center Metabolomics Facility (http://metabolomics-core.ucdavis.edu/; Fiehn et al., 2005). Metabolite identity was determined by comparing retention time and mass to the UC Davis Genome Center Metabolomics Facility metabolites database (http://fiehnlab.ucdavis.edu/Metabolite-Library-2007/; Fiehn et al., 2005). At the time of analysis, this library contained reference spectra for 1013 known metabolites, generated by the analysis of purified reference compounds. Metabolites not contained within this library are listed as unknown or unidentified metabolites using a unique database identifier (http://fiehnlab.ucdavis.edu/Metabolite-Library-2007/). For the metabolomics measurements in these experiments, this identified 439 putative metabolites within the entire experiment (Table S2). Post analysis, samples were subject to quality control and normalization as previously described using a pooled reference standard to control for technical variance (Fiehn et al., 2008; Fernie et al., 2011).

### Statistics

To test the effect of *MNM1* upon metabolite accumulation at the mid-day time point, all metabolites were analyzed via ANOVA using a general linear model within (R Development Core Team, 2014). First, a primary model was run to test the effect of general genotypes (i.e., WT v *mnm1-1/2* v OE7/8). In this model *y*_*rgc*_ denotes the metabolite accumulation in each plant with Genotype *g*, from Replicate *r*. The model for the metabolite accumulation is

$$y_{rgc}=\mu +G_{g}+R_{r}+\varepsilon_{rg}$$

where ε_*rg*_ represents the error term and is assumed to be normally distributed with mean 0 and variance σ^2^_ε_. All parameters are reported in Table S3. Another model was also used in which the individual *mnm1-1/2* and OE7/8 genotypes were nested within the broader genotype term. These two models were then compared for significant difference and this is reported in Table S3 for each metabolite. Means and standard error for each genotype class were obtained using (R Development Core Team, 2014). All presented p values are adjusted to FDR of 0.2. We have broadly used this FDR level in the past with a high level of validation (West et al., 2007; Rowe et al., 2008; Chan et al., 2011; Kerwin et al., 2011). Further, this is a similar level of significance to that used in the original QTL mapping.

To test the connection between genotype and time within the diurnal/circadian time course the same model approach was utilized to test for genotype × time interactions to obtain a precursory impression of time-specific genotype shifts. Given that this short time course has both transition and stable circadian time points because of the inclusion of the first day in the transition to constant light, this specific time testing is considered appropriate for the purpose of identifying genotypic effects upon plant metabolism. In this model *y*_*rgc*_ denotes the metabolite accumulation in each plant with Genotype *g*, from Time *t*, from Replicate *r*. The model for the metabolite accumulation is

$$y_{rgt}=\mu +G_{g}+T_{t}+R_{r}+G_{g}xT_{t}+\varepsilon_{rgt}$$

where ε_*rgt*_ represents the error and is assumed to be normally distributed with mean 0 and variance σ^2^_ε_. Means and standard error for each genotype class were obtained using (R Development Core Team, 2014). All presented p values are adjusted to FDR of 0.2 (Tables S2, S4). We have broadly used this FDR level in the past with a high level of validation (West et al., 2007; Rowe et al., 2008; Chan et al., 2011; Kerwin et al., 2011). Further, this is a similar level of significance to that used in the original QTL mapping.

To test the effect of Bay and Sha allelic complementation of the *mnm1-1* and *mnm1-2* genotypes, the following model was used:

$$y_{ragt}=\mu +G_{g}+A_{a}\left(G_{g}\right)+T_{t}+R_{r}+G_{g}xT_{t}+A_{a}\left(G_{g}\right)xT_{t}+\varepsilon_{ragt}$$

where ε_*rgt*_ represents the error and is assumed to be normally distributed with mean 0 and variance σ^2^_ε_. In this model, *y*_*rgc*_ denotes the metabolite accumulation in each plant with Allele *a*, Genetic background *g*, from Time *t*, from Replicate *r*. Allele represents the Bay and Sha alleles introduced into the *mnm1-1* and *mnm1-2* genetic background and is nested in genetic background. Means and standard error for each genotype class were obtained using (R Development Core Team, 2014). All presented *P*-values are adjusted to FDR of 0.2 (Tables S5, S6). We have broadly used this FDR level in the past with a high level of validation (West et al., 2007; Rowe et al., 2008; Chan et al., 2011; Kerwin et al., 2011). Further, this is a similar level of significance to that used in the original QTL mapping.

## Results

### Identification of a candidate gene for the Met.V.67 QTL

**Figure 1:**
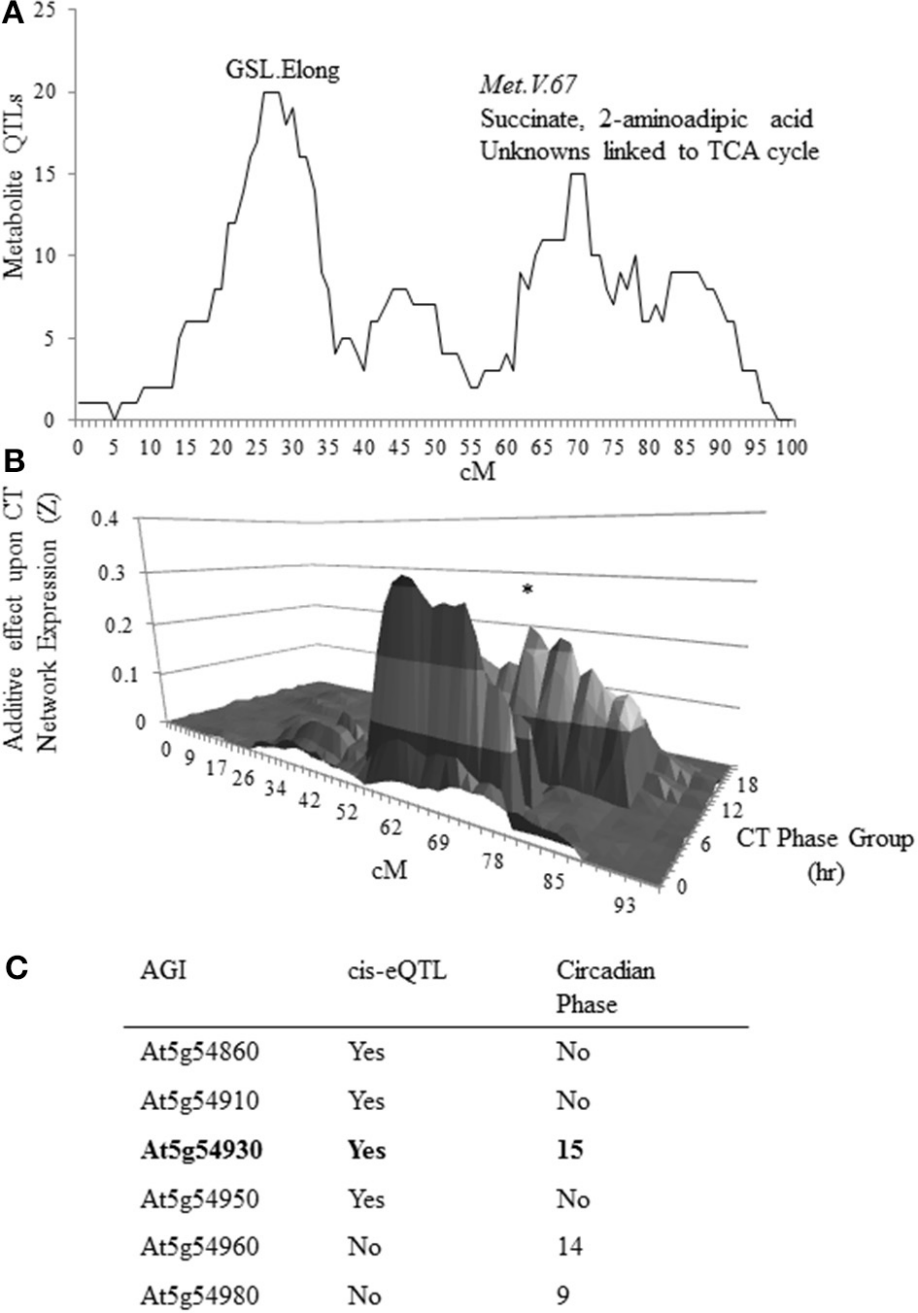
**Candidate identification for metabolomics QTL MET.V.67. (A)** The number of metabolites for which a QTL was detected in the Bay × Sha RIL population on chromosome V within a 10 cM sliding window is shown. The permuted threshold (*P* = 0.05) for detection of a significant metabolite hotspot within this analysis is 12 metabolite QTLs. The X axis shows the chromosomal position in cM. The known metabolomics QTLs on chromosome V are labeled as GSL.Elong and Met.V.67 with the known chemicals listed above the peak. **(B)** Shown is the impact of natural variation on chromosome V in the Bay × Sha RIL population upon expression of circadian CT phase group networks from previously published data. Each CT phase group consists of the genes that have a peak of circadian expression within 30 min of the time for that group. The graph presents the predicted additive effects across chromosome V for each CT phase group's expression from CT0 to CT23. The asterisk marks the CT phase group that is most impacted at the *Met.V.67* locus (CT15).The bottom shows the position on the chromosome in cM. **(C)** Shown is the list of candidate genes in the 3-LOD interval for the metabolomics QTL *Met.V.67* (At5g54830 to At5g55040) that have either a *cis*-eQTL or are regulated in a circadian manner with the phase reported.

To test whether we could identify genes underlying metabolomic QTLs by searching for genes that are affiliated with the circadian clock and contain a cis-eQTL, we focused on the Met.V.67 QTL that is linked to variation in both metabolite accumulation (Figure 1A) and circadian network output (Figure 1B). In the previous analysis the metabolic effect had been ascribed solely due to the Bay-0 allele but this is complicated by the inability of natural variation studies to ascribe an appropriate baseline. Instead, this requires cloning of the underlying gene. To accomplish this, we first defined the physical boundaries of Met.V.67 as the complete 3-LOD interval for all metabolites with a QTL peak at this position, as previously found (Rowe et al., 2008). Within this region, we surveyed all six genes for either a cis-eQTL or circadian oscillation using previous datasets (Figure 1C) (Harmer and Kay, 2005; Covington and Harmer, 2007; West et al., 2007; Covington et al., 2008). This analysis showed that only one gene, *At5g54930*, had both a cis-eQTL and a circadian expression pattern in agreement with the overlapping circadian network eQTL. This gene also tightly co-expressed with *TOC1* and *PRR5*, central components of the circadian oscillator (Figure S1) (Obayashi et al., 2007, 2009; Harmer, 2009). Thus, we hypothesized that *At5g54930* could be causative for the Met.V.67 metabolomic and circadian network QTL and renamed this gene *METABOLIC NETWORK MODULATOR 1*, *MNM1*.

### Genetic analysis of MNM1 variation

**Figure 2:**
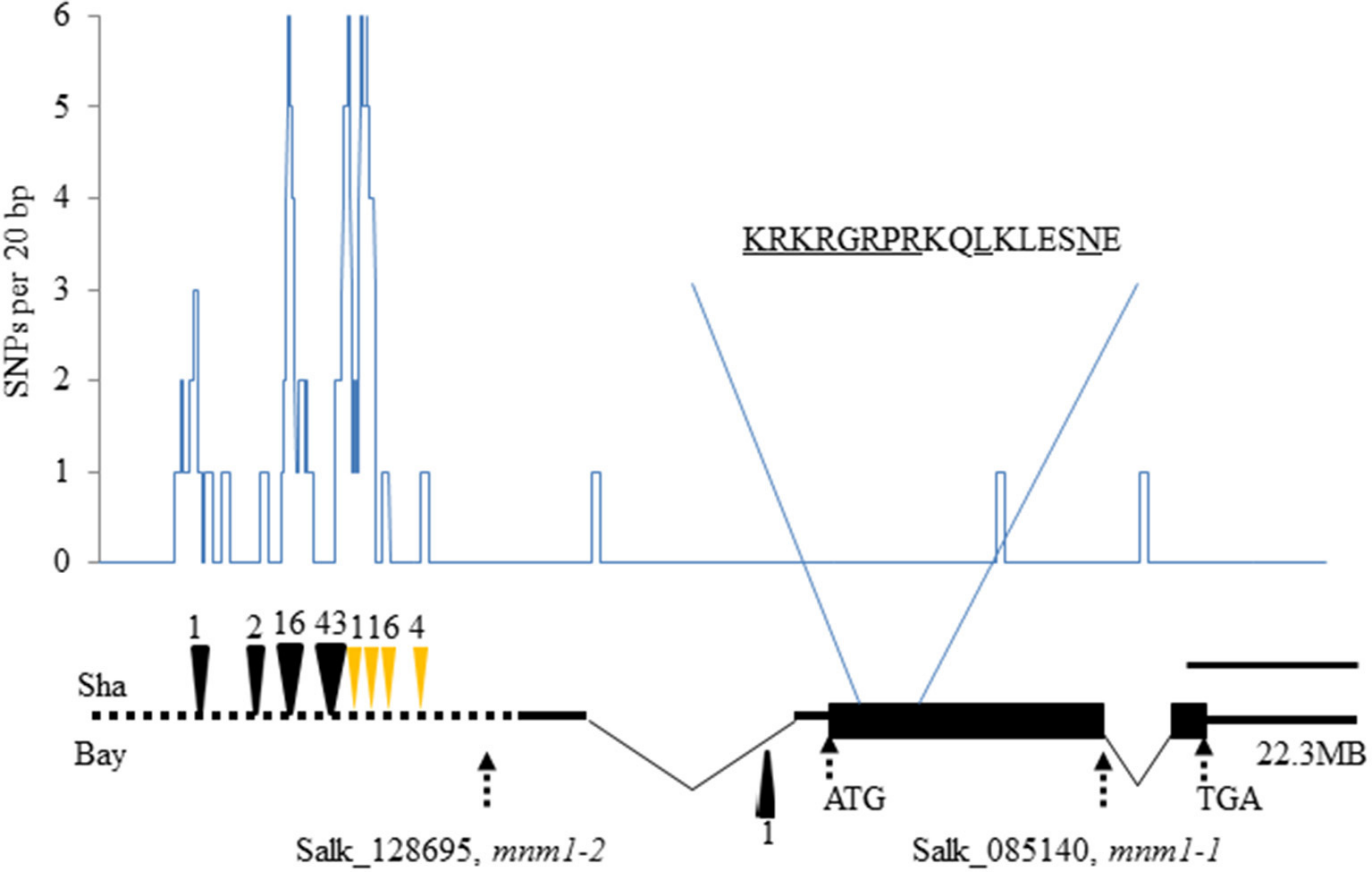
**Natural and induced variation in *MNM1***. The gene structure of *MNM1* is shown with the wide black rectangle showing the open reading frame. The 5′ and 3′ untranslated regions are shown as solid black lines with introns shown by the thin triangle. The promoter analyzed is shown by the dotted black line. The graph shows the number of single nucleotide polymorphisms per 20 basepairs between the Bay and Sha genomic sequences. The deletions with respect to the Col-0 sequence are shown as black triangles at the respective positions, while insertions are shown as yellow triangles. The position of *mnm1-2* (SALK_128695) and *mnm1-1* (SALK_085140) are presented. The scale represents a distance of 400 bp. The amino acid sequence shows the portion of the protein containing the AT-hook motif (RGRP), the potential nuclear localization sequence (KRKR) and the putative D-box (RxxLxxxxN). The start codon, stop codon, and chromosomal position are also labeled.

*MNM1* encodes a putative 286-amino-acid protein that contains an AT-Hook motif (RGRP; Figure 2). At-Hook motifs are often involved in binding DNA minor-grooves (Aravind and Landsman, 1998; Fujimoto et al., 2004) and can play roles in plant disease resistance, hormone metabolism, development, and other complex phenotypes (Matsushita et al., 2007; Ng et al., 2009; Gallavotti et al., 2011; Jin et al., 2011; Yadeta et al., 2011). The AT-hook motif was found close to a Degradation box and a nuclear localization sequence, but no other identifiable motifs were present in the protein sequence (Figure 2). Interestingly, MNM1 is not a member of the previously identified family of Arabidopsis AT-hook motif-containing proteins (Fujimoto et al., 2004). Instead, *MNM1* is a single copy gene within *Arabidopsis thaliana* and has a full-length homolog only in the Brassicales genomes of *Arabidopsis lyrata*, *Capsella rubella*, and *E. salsugineum*. All other tested eudicot genomes, such as *Brassica rapa*, *Vitis vinifera*, *Populus trichocarpa*, and *Ricinus communis*, have only partial paralogs that are related solely by the 80 amino acids surrounding the AT-hook motif (Figures S2, S3). This leads to an average identity of only 33% to the closest paralogs solely centered around the AT-hook motif (Figure S2 and Table S7). Thus, *MNM1* appears to be unique to the Brassicales lineage and it was apparently lost in the *Brassica* species (Figure S2).

To test if *MNM1* had the potential to be a causal basis of the Met.V.67 QTL we sequenced the gene in both Bay-0 and Sha parental accessions to look for genetic variation. This identified numerous polymorphisms within the *MNM1* promoter, including 40 SNPs and nine separate insertion/deletion events (Figure 2 and Figure S4). There were three SNPs within the predicted body of the RNA but these did not affect any key splicing sites and only a single Leu to Ile change was introduced into the protein. Thus, while it is more likely that the *MNM1* QTL is caused by the variation between the Bay and Sha promoters the amino acid change could potentially affect the protein function or the sequence change potentially alter RNA processing.

### Genetic analysis of MNM1 effect on arabidopsis physiology

Two T-DNA insertion lines from the Arabidopsis Biological Resource Center were obtained to further analyze the potential role of *MNM1* in Arabidopsis metabolism. SALK_085140, hereafter *mnm1-1*, contained a T-DNA in the open reading frame, whereas SALK_128695, hereafter *mnm1-2*, contained a T-DNA very near the transcription start site (Figure 2; Alonso et al., 2003). qRT-PCR of these alleles showed that *mnm1-1* leads to a 5-fold reduction in transcript abundance with complete ablation of the last exon (Figure S5). By contrast, *mnm1-2* had a 2-fold increase in transcript abundance above WT. Neither *mnm1-2* nor *mnm1-1* displayed altered expression of the neighboring *At5g54920* and *At5g54940* genes (Figure S6). We created *MNM1* overexpression constructs by fusing the coding sequence to a 35S promoter. Two independent T3 lines, OE7 and OE8, from independent T1 events that had homozygous insertions with minimal gross morphological effects were chosen for further analysis. These lines both exhibited a >10× increase in *MNM1* transcript levels (Figure S5).

**Figure 3:**
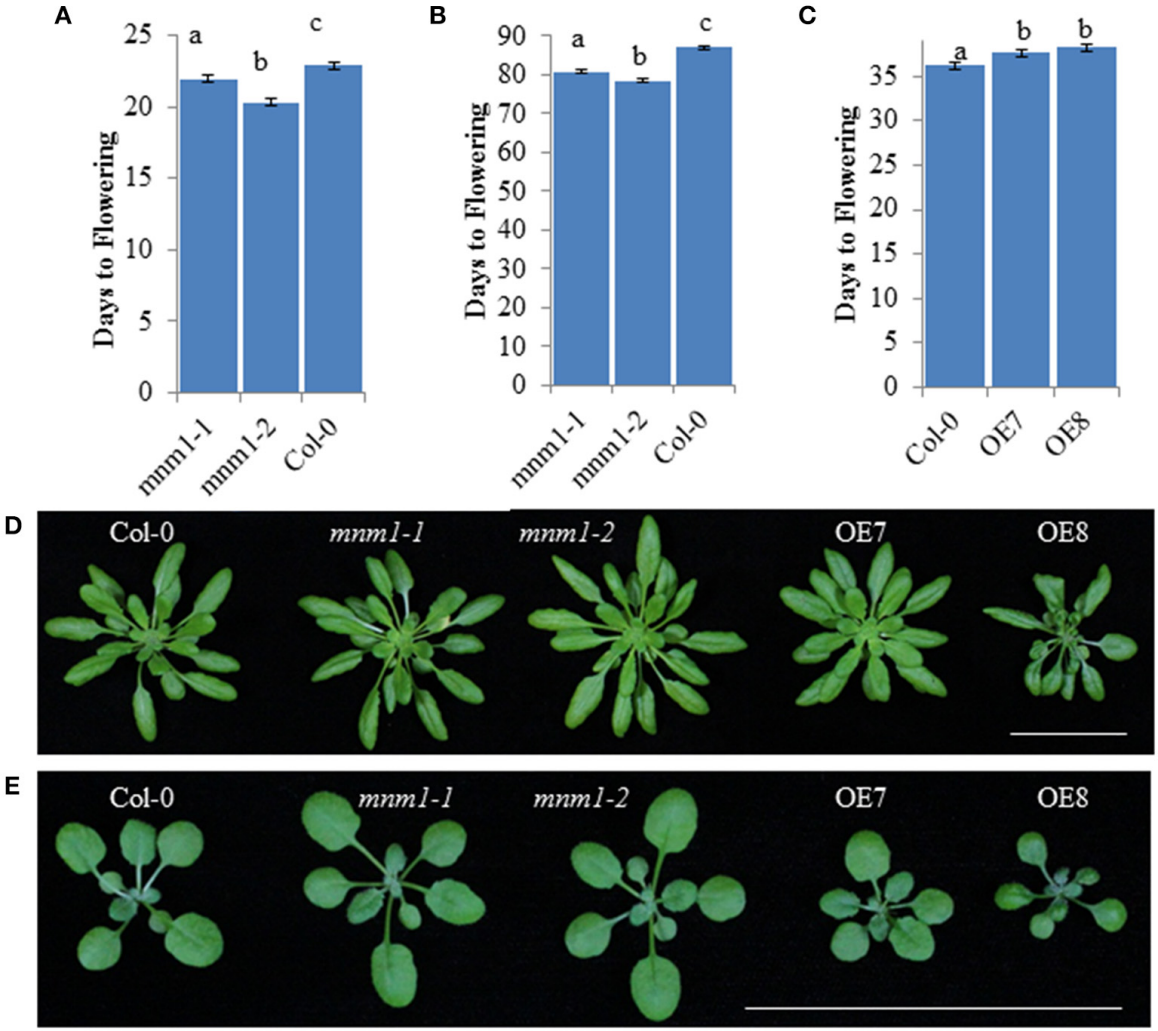
**Morphological analysis of *MNM1* Genotypes**. Letters show statistically different groupings within a graph as determined by ANOVA at a 0.001 level. All ANOVA models included genotype and the interaction of genotype and replicate. In all instances the interaction term was non-significant. All values are least squared means with standard error. For **(A,B)**, all genotypes were tested together in four independent experiments per day length with an average of 200 plants in total across the experiments for each genotype. For C, Col-0 and the two *MNM1* OE genotypes were tested in three replicates with 50–54 plants per genotype. **(A)** Number of days to flowering under long-day conditions for WT and the *mnm1-1* and *mnm1-2* mutants. **(B)** Number of days to flowering under short-day conditions for WT and the *mnm1-1* and *mnm1-2* mutants. **(C)** Number of days to flowering under long-day conditions for WT and two *MNM1* overexpression genotypes. **(D)** Growth of 6-week old plants under short-day conditions. The bar shows a 5 cm scale. **(E)** Growth of 3-week old plants under long-day conditions. The bar shows a 5 cm scale.

Using a nested ANOVA allowed us to test for differences between *MNM1* and *mnm1* as well as between the *mnm1* alleles. This showed that both *mnm1-1* and *mnm1-2* displayed slightly earlier flowering under both short and long-day conditions, suggesting that the T-DNA insertions similarly affect the flowering function of *MNM1* even with their different effects on expression of the transcript (Figure 3 and Figure S7, Table S8). This idea is supported by the finding that both OE lines had a slight delay in flowering (Figure 3), suggesting that the effects of high-level overexpression are opposite to those of slight overexpression or down-regulation of *MNM1* (Figure 3 and Figure S8). Approximately half of all obtained *MNM1* OE lines showed even more dramatic developmental consequences but these lines were not further analyzed because of the difficulty of separating the morphological and metabolic consequences of *MNM1* (Figure S8). Thus, the *mnm1-1* and *mnm1-2* alleles have similar physiological consequences that are the opposite of those observed in the OE lines, suggesting that *MNM1* plays a role in plant development. There are several possible explanations about why the *mnm1-1* and *1-2* alleles have similar physiological phenotypes while divergent transcript abundances. *mnm1-2* may have altered transcript processing, stability or translation which would disconnect RNA abundance from function. Additional work is required to understand the precise basis of the phenotypic cause in *mnm1-2*.

### Genetic analysis of MNM1 in arabidopsis metabolism

To test the link between *MNM1* and the Met.V.67 QTL, we measured metabolite accumulation in *mnm1-1* and *mnm1-2* and *MNM1* overexpression genotypes as well as the wild type Col-0 accession. The samples were harvested at mid-day in short-day conditions, which is the same time and light regime as the original metabolomic and expression QTL experiments used to identify the candidate gene (West et al., 2007; Rowe et al., 2008; Kerwin et al., 2011). 459 metabolites were detected and all were subjected to nested ANOVA at an FDR adjusted level of 0.2 (Table 1 and Table S3) (West et al., 2007; Rowe et al., 2008; Kerwin et al., 2011). We have broadly used this FDR level in the past with a high level of validation (West et al., 2007; Rowe et al., 2008; Chan et al., 2011; Kerwin et al., 2011). Further, this is a similar level of significance to that used in the original QTL mapping. Supporting the hypothesis that *MNM1* variation causes the Met.V.67 QTL, succinate was significantly altered in the OE lines in comparison to Col-0. Nested ANOVA again showed that the two *mnm1* alleles behaved identically with respect to each other and that the two OE lines were identical to one another (Table 1 and Table S3), this allows us to combine data from the recessive *mnm1* and dominant OE lines in further analysis.

**Table 1:** **Known metabolites altered at mid-day by *MNM1* genotypes**.

	**Wildtype**		***mnm1-1/2***		**OE**	
**Metabolite**	**Avg**	***SE***	**Avg**	***SE***	**Avg**	***SE***
**CENTRAL C/N METABOLITES**						
Hydroxylamine	10033	1392	14533*	1077	11588	1167
Allantoin	572	54	455*	32	606	61
Arginine + ornithine	1274	89	1602*	108	1274	82
Pyruvic acid	3083	490	5143*	700	4294*	683
Succinic acid	3876	232	3651	237	4536*	206
Glyceric acid	11368	1309	17464*	1664	15693*	1441
Enolpyruvate	433	41	658*	76	546*	64
**SUGARS**						
Rhamnose	7290	626	8235*	415	8394*	523
Sophorose	318	55	411*	25	351	27
Glycero-guloheptose	5660	692	6744*	469	6433*	505
N-acetyl-D-hexosamine	3922	410	4078	184	4605*	209
N-acetyl-D-mannosamine	12471	1627	15666*	1035	14475*	1190
**MISCELLANEOUS**						
1-hexadecanol	633	70	611	34	510*	44
FAD	194	26	254*	19	217	19
Ferulic acid	182	17	230*	17	191	20

Average metabolite accumulation across the insertional (mnm1-1 and mnm1-2) and OE genotypes. A asterisk after the average (Avg) and standard error (SE) for either the mnm1-1/2 or OE genotypes indicates that the specific metabolites significantly differ across both alleles within either the mnm1-1/2 or OE genotypes with respect to WT in the nested ANOVA analysis. Each genotype was measured in 8 fold replication across two independent experiments (N = 8 for WT, N = 16 for mnm1, and N = 16 for OE). Unknown compounds are listed in Table S3.

Comparison of Col-0 WT to the OE lines mimicked the QTL pattern of additive effects between the Bay and Sha alleles of Met.V.67. Specifically, the OE lines displayed higher accumulation of all compounds associated with the Met.V.67 QTL in comparison to Col-0, just as the Sha allele led to higher accumulation of these compounds in comparison to the Bay allele (Table S3). This is in accordance with previous observations that the Col-0 and Bay-0 alleles of *MNM1* are expressed at similar levels whereas the Sha allele displays higher expression (Kliebenstein et al., 2006; Van Leeuwen et al., 2007; West et al., 2007), and our current result that the genomic sequence of *MNM1* in Bay is more similar to Col-0 than Sha. Therefore, the comparison of *MNM1* OE lines with Col-0 better recreates the natural variation originally studied in the Bay-0 × Sha RIL population. Further supporting *MNM1* as the causative locus of the Met.V.67 QTL, none of the metabolites linked to QTLs flanking Met.V.67 (±10 cM) were altered by the various *MNM1* genotypes (Table S3).

The specific manipulation of *MNM1* within an isogenic background provides us more statistical power and genetic precision to fully interrogate the effects of *MNM1* upon plant metabolism in comparison to the previous Bay-0 by Sha QTL analysis. Therefore, we analyzed the full metabolomics data to identify any additional metabolites that might be linked to alterations in *MNM1*. ANOVA of all 459 detected metabolites showed that 49 metabolites were significantly altered by the differences in the *MNM1* genotypes (Table S3). Of these, 38 metabolites did not show a significant nesting term when comparing *mnm1-1* and *mnm1-2* to each other as well as the OE alleles to each other, indicating that *mnm1-1* and *mnm1-2* as well as OE7 and OE8 behaved similarly to each other in terms of those metabolites (Table S3). Surprisingly, levels of only six of the known metabolites were statistically altered in both the OE and *mnm1-1/2* lines and in these cases the directionality of the change was always the same in both groups of lines (Table 1). This directional agreement between the *mnm1-1/2* and OE genotypes was also true for unidentified metabolites in this study (Table S3, Figure S9). This similarity in effect of both OE and *mnm1-1/2* upon steady-state metabolite accumulation suggests that *MNM1* may play a role in determining metabolic balance rather than any specific directional effect. The discrepancy wherein the OE and *mnm1-1/2* lines display opposite flowering time phenotypes despite similar metabolic alterations suggests that these metabolic and physiological outcomes may reflect independent functions of *MNM1*. Alternatively, there may be an as-yet unmeasured aspect of metabolism, such as flux, that is more directly linked to the physiological consequences of *MNM1* activity. Future experiments are necessary to resolve this interesting observation.

### Genetic analysis of *MNM1* in arabidopsis metabolic balance

**Figure 4:**
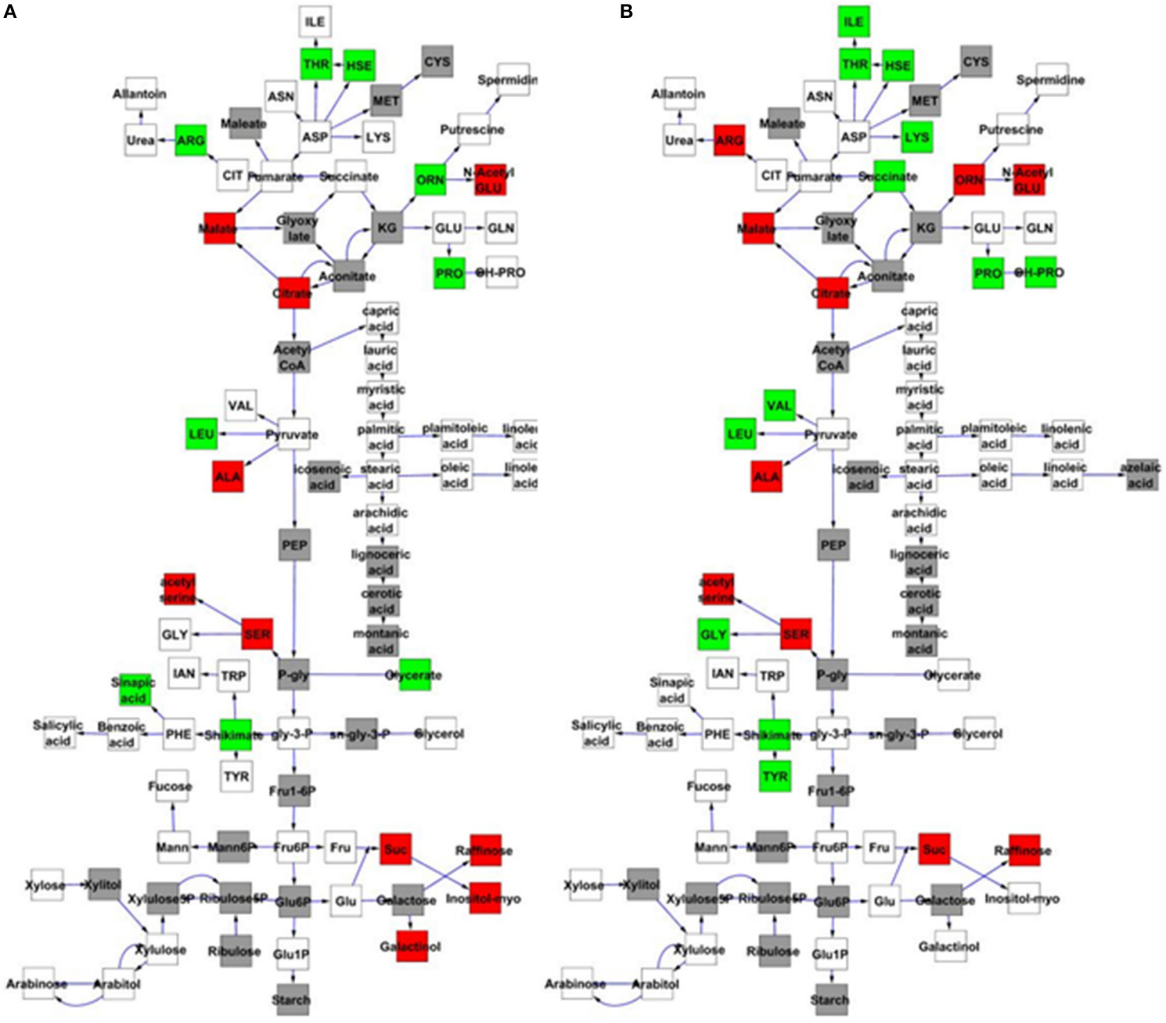
***MNM1* effects upon the metabolic network**. Shown are the known primary metabolites that are altered at all timepoints by the *mnm1-1* and *mnm1-2* and overexpression genotypes in comparison to wildtype controls within the free-run experiment. The gray boxes show metabolites not measured in these experiments, white boxes are metabolites that had no significant difference, green boxes were up regulated in the respective *MNM1* genotypes and red boxes were down-regulated in the respective *MNM1* genotypes. Only metabolites showing a significant effect across the *mnm1-1* and *mnm1-2* and OE genotypes are presented. The central metabolites are presented in a stylized representation of central metabolism with common abbreviations for each chemical. Arrows represent enzymatic linkages between detected compounds but are not necessarily showing single enzymatic steps. All detected intermediates such as shikimate are included in the network. **(A)** Metabolites altered in both the *mnm1-1* and *mnm1-2* genotypes. **(B)** Metabolites altered in both *MNM1* OE genotypes.

To further examine the effect of *MNM1* on metabolic homeostasis within Arabidopsis, we analyzed metabolite accumulation in the *mnm1-1/2* and OE lines across a 48 h time course. Given that *MNM1* was linked to circadian clock output (Figure 1), we left the plants in constant light for the entire time course to get a preliminary view of how the genetic polymorphisms within *MNM1* affect metabolic homeostasis. 459 metabolites were detected and all were subjected to nested ANOVA (Tables S2, S4). This identified 122 metabolites that accumulated to significantly different levels in both *mnm1-1/2* or both OE lines in comparison to the WT genotype. Of the metabolites significantly different between the *MNM1* mutant alleles and the WT genotype, 96 showed no significant difference when comparing *mnm1-1* to *mnm1-2* or OE7 to OE8, suggesting that these metabolites show identical shifts upon any perturbation of *MNM1* (Tables S2, S4). While the vast majority of these metabolites showed significant variation across the time course, the genotypic influence was the same across all timepoints as indicated by the Genotype:Time interaction (Tables S2, S4). The known metabolites that were affected by the *mnm1-1/2* or OE genotypes at all timepoints are shown in a map of central metabolism (Figure 4). These metabolic effects were spread across the TCA cycle and general amino acid metabolism, but had no apparent effect on lipid metabolism (Figure 4). In both the *mnm1-1/2* and OE genotypes there seemed to be a rebalancing of metabolite accumulation away from TCA and sugars toward specific amino acids.

In central metabolism, ornithine was the sole exception to the finding that any perturbation of MNM1 gave rise to identical metabolic consequences. For ornithine, the *mnm1-1/2* genotypes led to higher accumulation whereas the OE genotypes had lower accumulation in comparison to WT. Outside of central metabolism, nine unknown metabolites and methionine sulfoxide, the position of which within the metabolic network is unknown, showed a similar pattern to ornithine, while glycerate had the same trend but was not significantly different in the OE background (Figure 4 and Tables S2, S4). By contrast, only three unknown metabolites showed the opposite pattern, with significantly higher accumulation in the *mnm1-1/2* lines and the reduced accumulation in the OE lines (Tables S2, S4). Thus, genetic alterations in *MNM1* lead to linear changes in accumulation for some metabolites, while for the other metabolites *MNM1* behaves more as a homeostasis controller whereby any change in MNM1 function leads to identical fluctuations in these metabolites (Figure 4). These data suggest that the flowering time phenotype in the MNM1 lines may be more linked to ornithine, methionine sulfoxide or any of the unknown compounds that are linearly related to MNM1 function.

**Figure 5:**
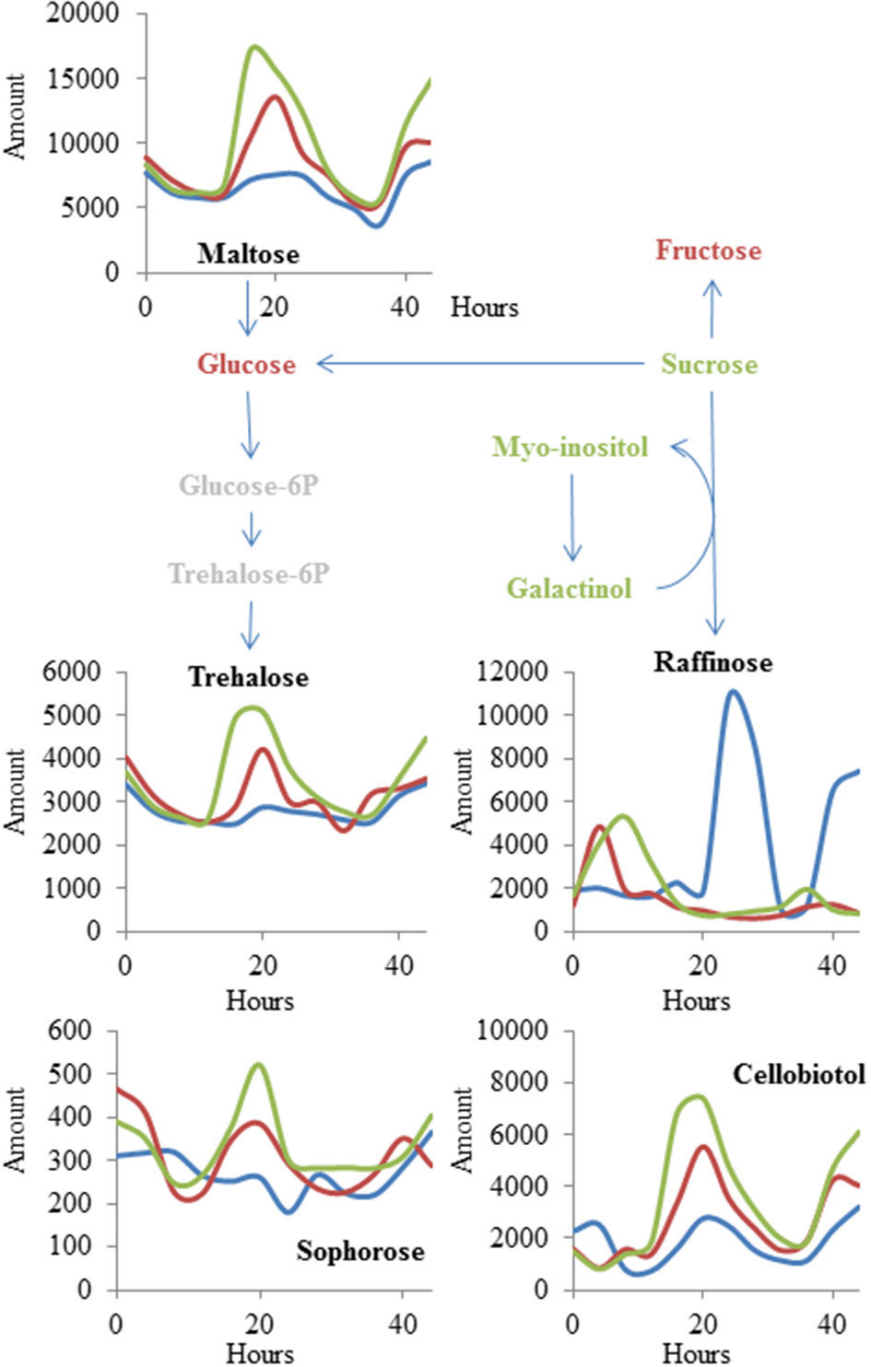
***MNM1* effects upon cycling within the metabolic network**. Shown are the known metabolites that were altered in a time-dependent fashion by the *mnm1-1*, *mnm1-2*, and *MNM1* OE genotypes in comparison to wildtype controls within the free-run experiment. The metabolites are placed within a sugar network for illustration. Red metabolites had no significant effect of *MNM1* function, green metabolites had a significant effect of *MNM1* function but no interaction with time and are shown in Figure 4. Metabolites labeled in black had a significant interaction of *MNM1* function and time within the analysis. Their levels are shown across the 44 h of the experiment in relative abundance levels obtained from the GC-TOF analysis. Blue lines in the graph represent WT Col-0, Red lines are the effect of *mnm1-1* and *mnm1-2*, while green is the effect of the OE7 and OE8 genotypes.

There was a small collection of metabolites for which the genotype: time interaction terms had more significance than the genotype main effect term (Tables S2, S4). This was most pronounced for a set of sugars (maltose, trehalose, raffinose, cellobiotol, and sophorose) (Figure 5). Both the OE and *mnm1-1/2* insertion lines show a diminished and shifted oscillation in free raffinose accumulation with a concomitant shift toward higher accumulation of the other sugars. These sugars are connected by the conversion of raffinose to glucose via sucrose, and the other sugars in this pathway (sucrose, galactinol, and myo-inositol) are all also decreased in the OE and T-DNA insertion lines. This suggests that *MNM1* plays a role in mediating the temporal partitioning of sugars within the Arabidopsis leaf. Further experiments will be required to test which is the most immediate effect on the metabolomics network of polymorphisms within *MNM1.*

### Quantitative complementation of *MNM1*

The above experiments demonstrated that *MNM1* alters metabolism and physiology of Arabidopsis in a manner similar to phenotypes that are linked to variation at the Met.V.67 metabolomic QTL. To validate that variation in the Bay and Sha alleles of *MNM1* were potentially causal for the Met.V.67 QTL, we cloned the full length-genomic sequences of *MNM1* from both the Bay and Sha genotypes and independently transformed these into both the *mnm1-1* and *mnm1-2* backgrounds. Comparison of two opposing alleles in a common genetic background is the definitive test for establishing that the alleles differ in their function (Mackay, 2001). Given that the *mnm1-1* and *mnm1-2* lines had similar phenotypes in terms of both the metabolite and flowering time data, we obtained independent homozygous transgenic lines containing either the Bay or Sha genomic allele in both backgrounds. We then measured metabolites in these complementation lines at 6 weeks of age at either 4 or 24 h past dawn. Four hours is the time point equivalent to that used for the QTL mapping sample, whereas 24 h was the time point that showed the most differences in the metabolomics time course (Figures 4, 5). Of the 11 metabolites originally detected as being altered by the Met.V.67 QTL, nine were detected in this experiment, succinate, 2-aminoadipic acid and seven unknowns (211891, 214529, 204348, 200450, 208664, 212373, and 213330). Only two unknown compounds were not found in this analysis. To test for quantitative complementation differences between the Bay and Sha *MNM1* alleles, we used an ANOVA that specifically tested all metabolites for differences between the transgenic plants containing the Bay and Sha *MNM1* alleles while controlling for any potential difference between the *mnm1-1* and *mnm1-2* backgrounds. This showed that the accumulation of eight of the nine metabolites linked to the Met.V.67 QTL was statistically different between the quantitative complementation lines containing the Bay or Sha allele (Tables S5, S6). In all instances, the direction of effect agreed with the previously published analysis from Met.V.67 (Tables S5, S6) (Rowe et al., 2008). The only exception was the unknown 212373. Thus, variation between the Bay and Sha alleles of *MNM1* is responsible for the vast majority of metabolomic variation at the Met.V.67 QTL in the Bay × Sha RIL population.

**Figure 6:**
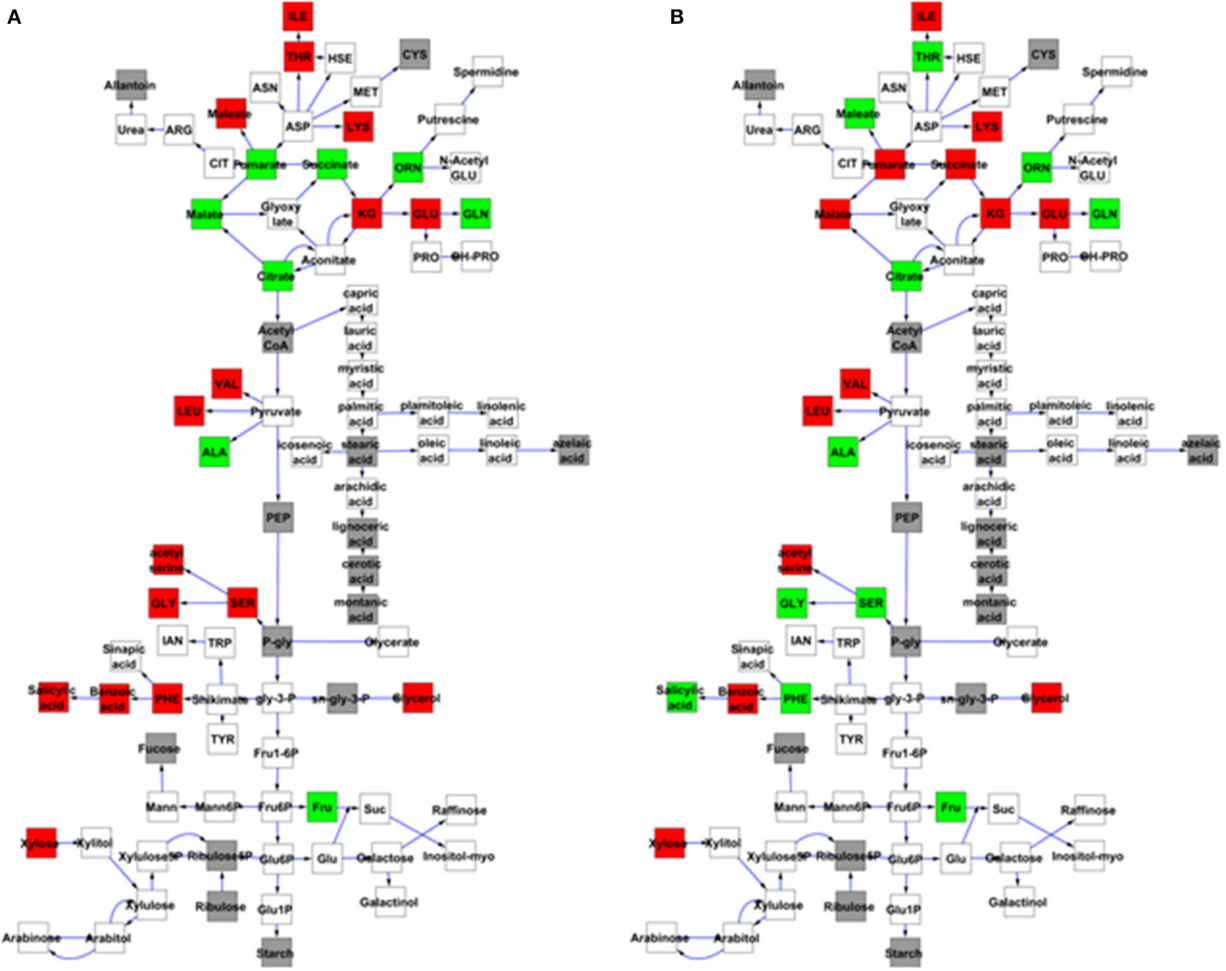
**Quantitative complementation to validate *MNM1* as the Met.V.67 QTL**. Full genomic Bay or Sha alleles of *MNM1* were transgenically reintroduced into both the *mnm1-1* and *mnm1-2* background genotypes. Homozygous T2 lines were analyzed for metabolomics at either 4 or 24 h in a free-run experiment as identified as key timepoints for MNM1 function. The 4 h timepoint is the same that was sampled to identify the Met.V.67 QTL. The effect on only central metabolites showing a significant difference between the Bay and Sha allele when introduced into both the *mnm1-1/2* genotypes presented at the specific timepoint shown. The gray boxes show metabolites not measured in these experiments, white boxes are metabolites that had no significant difference, green boxes are metabolites higher in Bay allele complementation lines and red boxes are metabolites higher in the Sha allele complementation lines. The metabolites in central metabolism are presented in a stylized representation of central metabolism with common abbreviations for each chemical. Arrows represent enzymatic linkages between detected compounds but are not necessarily showing single enzymatic steps. All detected intermediates such as shikimate are included in the network. **(A)** Metabolites that significantly differ between the Bay and Sha *MNM1* alleles at the 4 h timepoint. **(B)** Metabolites that significantly differ between the Bay and Sha *MNM1* alelles at the 24 h timepoint in constant light.

Most of these metabolites showed a genotype by time interaction, i.e., the Bay and Sha alleles had time-dependent effects on metabolite levels. This was particularly striking in the TCA cycle where succinate, fumarate, and malate shifted from the Bay allele conferring higher accumulation at the 4 h time point to the Sha allele leading to higher accumulation at the 24 h time point (Figure 6 and Tables S5, S6). Glycine, serine, phenylalanine, threonine and salicylic acid also displayed a time-dependent allelic effect. By contrast, other metabolites like glutamate and glutamine display more consistent behavior, with plants harboring the Bay and Sha alleles showing the same relationship at both time points. This suggests that the *MNM1* allelic variation underlying the Met.V.67 QTL has different temporal effects upon the metabolomics network.

### Expression analysis of *MNM1*

**Figure 7:**
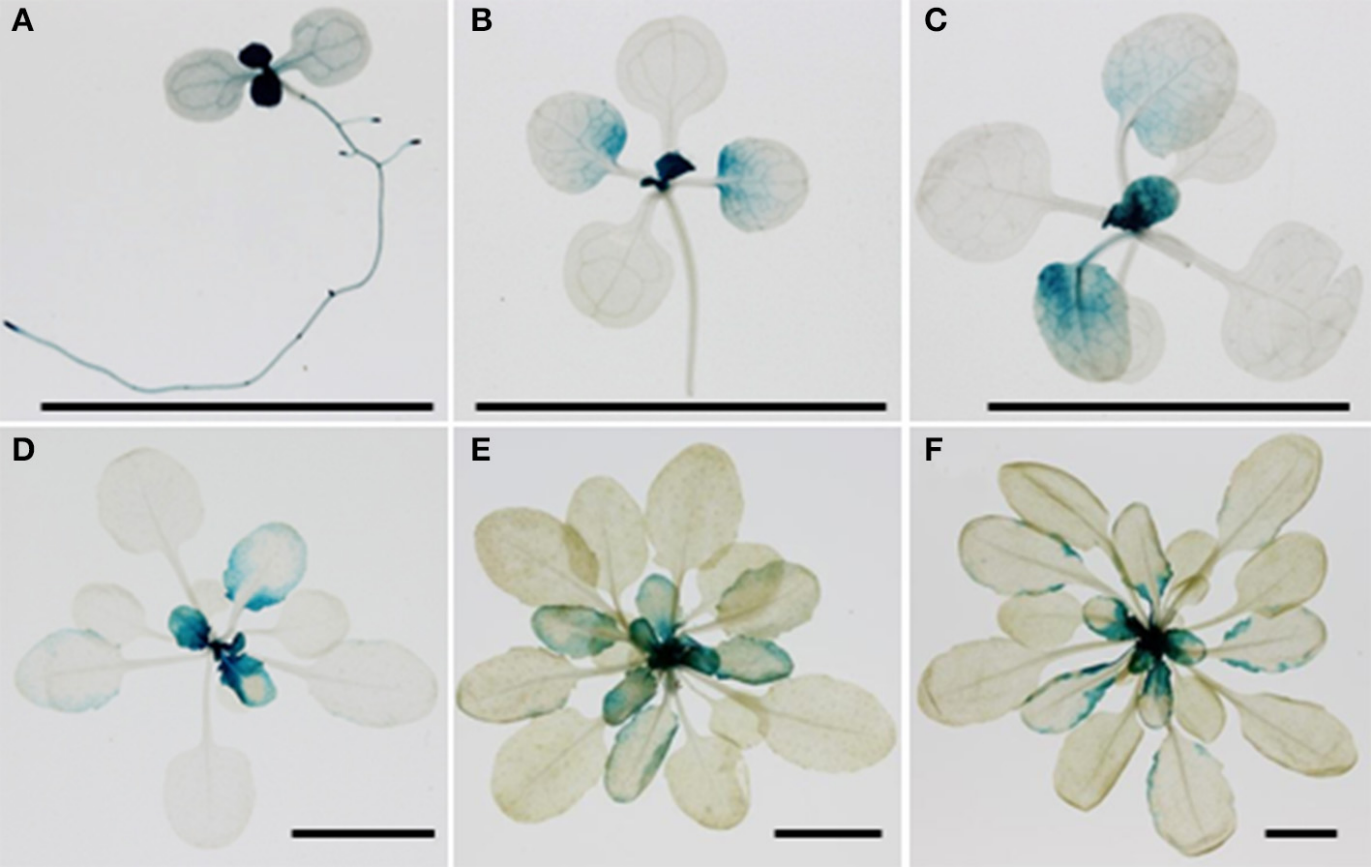
**Developmental patterning of *MNM1***. Shown is histochemical GUS staining of transgenic Arabidopsis plants expressing the *MNM1* translational fusion from the Sha accession in the Col-0 background. Three independent transgenic lines per construct were visualized for each time point and a representative image is shown. The black bar in each graph represents a 1 cm scale. **(A)** One-week-old seedling grown in MS medium in long day condition. **(B)** Two week-old rosette leaves grown in soil in short day condition. **(C)** Three week-old rosette leaves grown in soil in short day condition. **(D)** Four week-old rosette leaves grown in soil in short day condition. **(E)** Five week-old rosette leaves grown in soil in short day condition. **(F)** Six week-old rosette leaves grown in soil in short day condition.

The temporal aspect of the effect of *MNM1* allelic variation and the preponderance of natural variation in the promoter of *MNM1* led us to test for expression difference of the Bay and Sha alleles of *MNM1*. To do this, we cloned the genomic copies of *MNM1* from Bay and Sha and used these to make 3′ translational GUS-GFP fusions. Both the Bay and Sha GUS-GFP translational fusions were transformed into the Col-0, *mnm1-1* and *mnm1-2* backgrounds. Three to four independent T1s were chosen for each genotype/transgene combination and allowed to self-pollinate; then, three homozygous T3 plants from independent T1 lines were analyzed for developmental expression patterning of the two alleles of *MNM1.* GUS staining was performed once a week for 6 weeks post-germination in short-day conditions and once every week for 4 weeks post-germination in long-day conditions. There was no detectable difference between the expression patterns of the Bay and Sha alleles under any condition in any background in any of the independent transgenic lines (Figures S10–S12). At 1-week post germination, *MNM1* was most highly expressed in the newly emerging leaves and in the vasculature of the root and cotyledons (Figure 7). By 2-weeks post germination, the vascular expression was largely gone while the newly emerging leaves showed the highest expression. In the maturing leaves of these plants, *MNM1* was expressed at the base and excluded from the vasculature (Figure 7). During the development of short day plants, the newly emerging leaves always showed high expression of *MNM1* but as these leaves matured, the expression was gradually lost in the lamina until only the margin showed any *MNM1* expression (Figure 7). Finally, all detectable *MNM1* expression was lost in the most mature leaves. Thus, it does not appear that the variation in the Bay and Sha promoters of *MNM1* leads to altered developmental patterning of the translational fusion. Further work will be required to ascertain the mechanism by which variation in these two alleles leads to the altered metabolic networks.

## Discussion

This work identifies *MNM1* as a causal gene behind the Met.V.67 QTL that determines natural variation in primary metabolism. The Met.V.67 metabolomics QTL in the Bay × Sha RIL population was linked to variation in TCA cycle metabolites, such as succinate, as well as altered expression of genes that peak during a circadian cycle (Rowe and Kliebenstein, 2008; Kerwin et al., 2011). To elucidate the molecular basis of this locus, we used a systems interrogation of genes within the region of the Met.V.67 QTL and found a single gene, *MNM1*, which had variable expression in the Bay × Sha population and was expressed at the proper time of the circadian oscillation (Figure 1). We validated the effect of MNM1 on natural variation using quantitative complementation to compare the phenotypic effects of introducing the Bay and Sha genomic alleles of *MNM1* into two independent T-DNA insertion backgrounds. The quantitative complementation showed that variation within the Bay and Sha alleles led to the genotypes containing these transgenes to have identical changes in the steady state accumulation of eight of nine metabolites linked to variation in this locus (Figure 6) (Rowe and Kliebenstein, 2008). The availability of these isogenic lines containing only *MNM1* variation also allowed us to show that there were a large number of other metabolites affected by this variation (Figure 6). Thus, the Bay and Sha alleles of *MNM1* confer differential accumulation of metabolites associated with the Met.V.67 QTL, demonstrating that allelic variation in *MNM1* in part underlies the Met.V.67 QTL. It remains to be tested if additional genes within this region also contribute to the Met.V.67 QTL.

The most likely causal polymorphisms between the Bay and Sha alleles of *MNM1* are within the promoter region. Large indels were found when comparing the two promoters (Figures 2 and Figure S4). This agrees with the gene having a cis-eQTL in the Bay and Sha population, suggesting that there is a promoter polymorphism leading to differential expression of the two alleles (West et al., 2007). However, all of our efforts to identify differential expression between the Bay and Sha translational fusions failed to reveal a dramatic difference between the two alleles (Figure 7). The cis-eQTL for this gene represents a quite modest effect of only 15% (West et al., 2007), and it is possible that a difference this small would not be apparent in the translational GUS fusion assays. Additionally, the eQTL analysis was performed using whole rosette tissue and as such, there may be tissue or even temporal aspects to the cis-eQTL that we have not yet identified (West et al., 2007). It is also possible that the non-synonymous change in the protein may also be responsible for the allelic difference possibly due to differential translation, RNA metabolism or subtle effects on protein structure. Further work is required to identify the specific difference between the Bay and Sha alleles that leads to the alterations in plant metabolism (Figures 6, 7).

### *MNM1* alters metabolic networks both temporally and non-temporally.

The allelic effect of the Bay vs. Sha alleles of *MNM1* was temporally dependent, with the Bay and Sha alleles having opposite effects at the different time points (Figure 6). For example, the Bay allele had generally higher TCA metabolites at relative noon but lower levels of some amino acids compared to the Sha allele. This pattern then switched at the 24 h time point (Figure 6). Interestingly, other metabolites, such as glutamate and glutamine, were affected by alterations in *MNM1*, but these effects were not dependent on the sampling time. Thus, *MNM1* has the capacity to regulate primary metabolism homeostasis with both temporal and non-temporal effects depending upon the specific metabolite being measured. The temporally conditional effects agree with the linkage between *MNM1*, Met.V.67 and the circadian clock. However, it remains an open question how manipulating a single gene can lead to both stable and temporally conditional effects within the interconnected metabolomics network.

A conundrum of the work is that the over-expresser and knockout lines have similar phenotypic consequences upon the metabolic network. Given that *MNM1* is not likely an enzyme, the metabolic changes are likely not the direct result of *MNM1* activity but instead a consequence of the direct target of *MNM1*. One could suppose that this might be a consequence of a secondary stress situation. However, none of the common stress related metabolites (Salicylate, Proline, OH-Proline, and polyamines) show changes in the *MNM1* genotypes. Further, the *mnm1-1* which has little to know RNA has the same effect suggesting that this is not the result of MNM1 mis-expression in the wrong place or time. Thus, it suggests that if MNM1 is high or low there is the same metabolic output which is contrary to the typical gene where the function is correlated to the output. While there are very few biological examples of this function, the behavior of MNM1 to the metabolome is very similar to electrical circuits that display a XOR or XNOR function (Bonnet et al., 2013). In both XOR and XNOR circuits, high and low levels of input (MNM1 activity) give rise to one level of output (metabolite) while middle levels of input give rise to a different output. Thus, it is possible that the MNM1 gene functions within a XOR or XNOR style genetic circuit that can influence the metabolome. It will require cloning other genes surrounding MNM1 to test this possibility.

### Could *MNM1* be a controller of sink-source relations?

The metabolomics analysis showed that *MNM1* affects steady state metabolite accumulation in fully mature leaves of 6-week-old plants (Figures 4–6). For these plants, we specifically sampled the lamina at the widest point of the leaf, avoiding the edge and vasculature. Interestingly, all translational and transcriptional GUS fusions showed that at this age, *MNM1* is restricted to the young leaves and the edge of older leaves. As such, the tissue sampled for metabolomics has no evidence of expression of *MNM1*. This suggests that either MNM1 is transported as a protein or that there is some other mechanism for how this gene influences leaf metabolite profiles. One suggestion comes from the observation that raffinose levels across time are altered in genotypes that affect *MNM1* function. Raffinose is a key sugar in phloem transport that plays a central role in moving energy between source and sink tissues in plants (Lalonde et al., 2003). The fact that altering *MNM1* function leads to a dramatic reduction in raffinose in combination with the trend of *MNM1* expression in what should be sink tissues raises the possibility that *MNM1* plays a role in coordinating sink and source tissues with regard to the movement of energy or metabolites (Figures 5, 7).

A role in determining energy or metabolite homeostasis between source and sink tissues could be one possible explanation for how altering *MNM1* affects metabolite accumulation in tissues that do not appear to express this protein or transcript. It is likely that metabolism is not cell autonomous but instead is linked throughout the plant via the transpiration stream and movement of metabolites between source and sink tissues (Zhang et al., 2010). This potential role for *MNM1* in modulating metabolite flows within the plant could also explain why the different *MNM1* genotypes lead to altered physiological performance as measured by flowering time and biomass accumulation (Figure 3). The tissue-specific pattern of expression for this gene means that future efforts to describe its regulatory mechanism should focus on newly emerging leaves or leaf edges, tissues that are not frequently investigated.

### How does a novel gene obtain the ability to affect primary metabolism?

Our results suggest that *MNM1* has the capacity to regulate central metabolism homeostasis and consequently alter growth and flowering time in *Arabidopsis thaliana*, potentially from within sink tissues. The ability to regulate source/sink relations is a fundamental physiological process that is shared by all plants. This would then lead one to expect that genes controlling this shared process should be present in all plant species, yet all of our bioinformatics analysis suggests that *MNM1* is a gene unique to the Brassicales lineage as we cannot find a close homolog within other eudicot genomes. Other genomes contain paralogs that are more closely related to different Arabidopsis genes (Figure S2). Thus, it appears that the Brassicales lineage has derived a new gene with the ability to influence a core physiological process. This raises the intriguing question of how a new gene can arise and intercalate into the regulatory network for a core physiological process without disrupting it. Similarly, if this can occur once in a plant lineage it could happen in other lineages. The modern reliance on focusing upon homologs of genes with known function leads to an ascertainment bias against studying unique genes, and as such there is no way of currently assessing how frequently conserved physiological processes may have lineage-specific factors. The fact that *MNM1* was found through studying natural variation suggests that if these variable lineage-specific factors exist in other plants and crops that they could be playing an unrecognized role in domestication or adaptation. At the very least, this suggests that more effort needs to be put into understanding how lineage-specific genes may link to non-lineage specific physiology.

## Conclusion

Our quantitative complementation shows that *MNM1*, a gene unique to the Brassicales, is likely a causal gene for the metabolomics QTL Met.V.67 in the Arabidopsis Bay × Sha RIL population. This demonstrates that genes can be identified solely through their ability to alter metabolomics variation. Analysis of gene expression and metabolomics effects lead us to hypothesize that *MNM1* may modulate source/sink relations in the plant. Little is known about how plants control source/sink relations or how they control metabolic networks in a whole-plant level; however, *MNM1* provides a unique tool to begin investigating these questions central to plant metabolism. Future experiments will be required to identify the specific molecular mechanism by which *MNM1* generates the observed metabolomics and physiological whole plant effects. Similarly, future work is required to investigate how often lineage-specific genes may influence phenotypes previously thought to be conserved across diverse lineages.

### Conflict of interest statement

The authors declare that the research was conducted in the absence of any commercial or financial relationships that could be construed as a potential conflict of interest.
