# Supplementary material for: The AT-hook motif-encoding gene METABOLIC NETWORK MODULATOR 1 underlies natural variation in Arabidopsis primary metabolism
Source: Front Plant Sci. 2014 Aug 22;5:415. doi: 10.3389/fpls.2014.00415 (PMC4141330; doi:10.3389/fpls.2014.00415)
Supplement: Supplementary file 1 [file Presentation1.PPTX]

## Slide 1
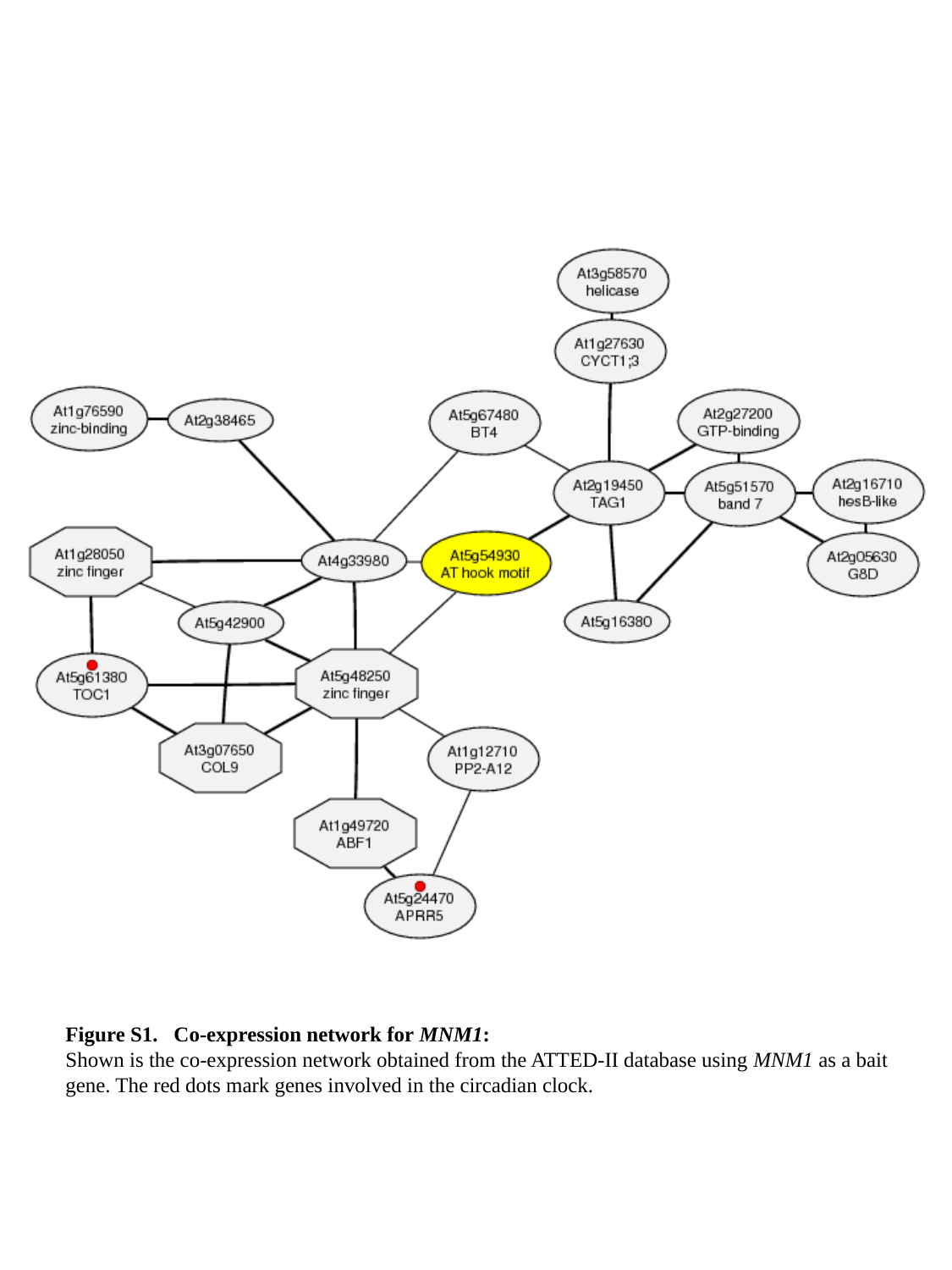

# Figure S1. Co-expression network for MNM1: Shown is the co-expression network obtained from the ATTED-II database using MNM1 as a bait gene. The red dots mark genes involved in the circadian clock.

## Slide 2
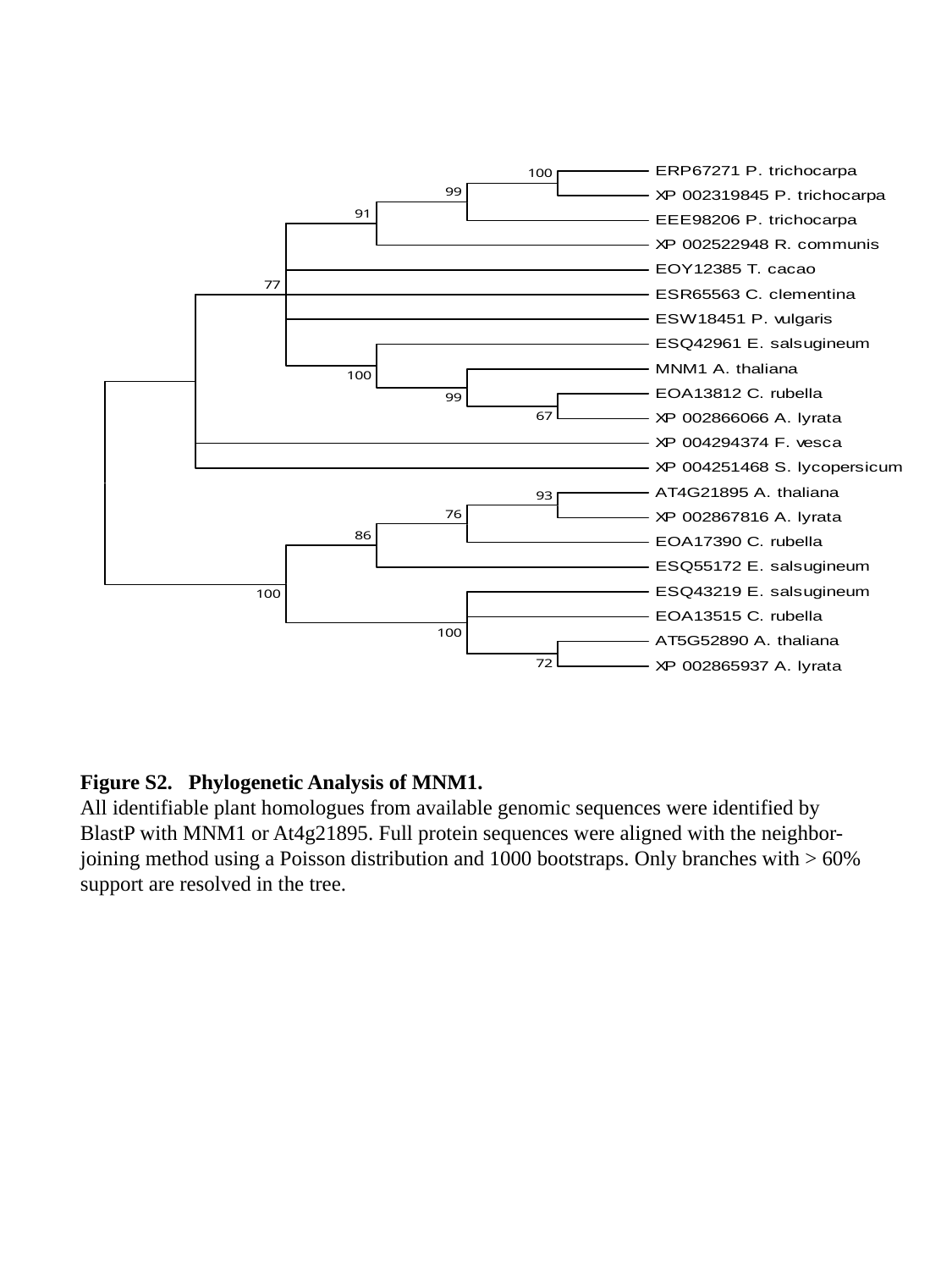

Figure S2. Phylogenetic Analysis of MNM1.
All identifiable plant homologues from available genomic sequences were identified by BlastP with MNM1 or At4g21895. Full protein sequences were aligned with the neighbor-joining method using a Poisson distribution and 1000 bootstraps. Only branches with > 60% support are resolved in the tree.

## Slide 3
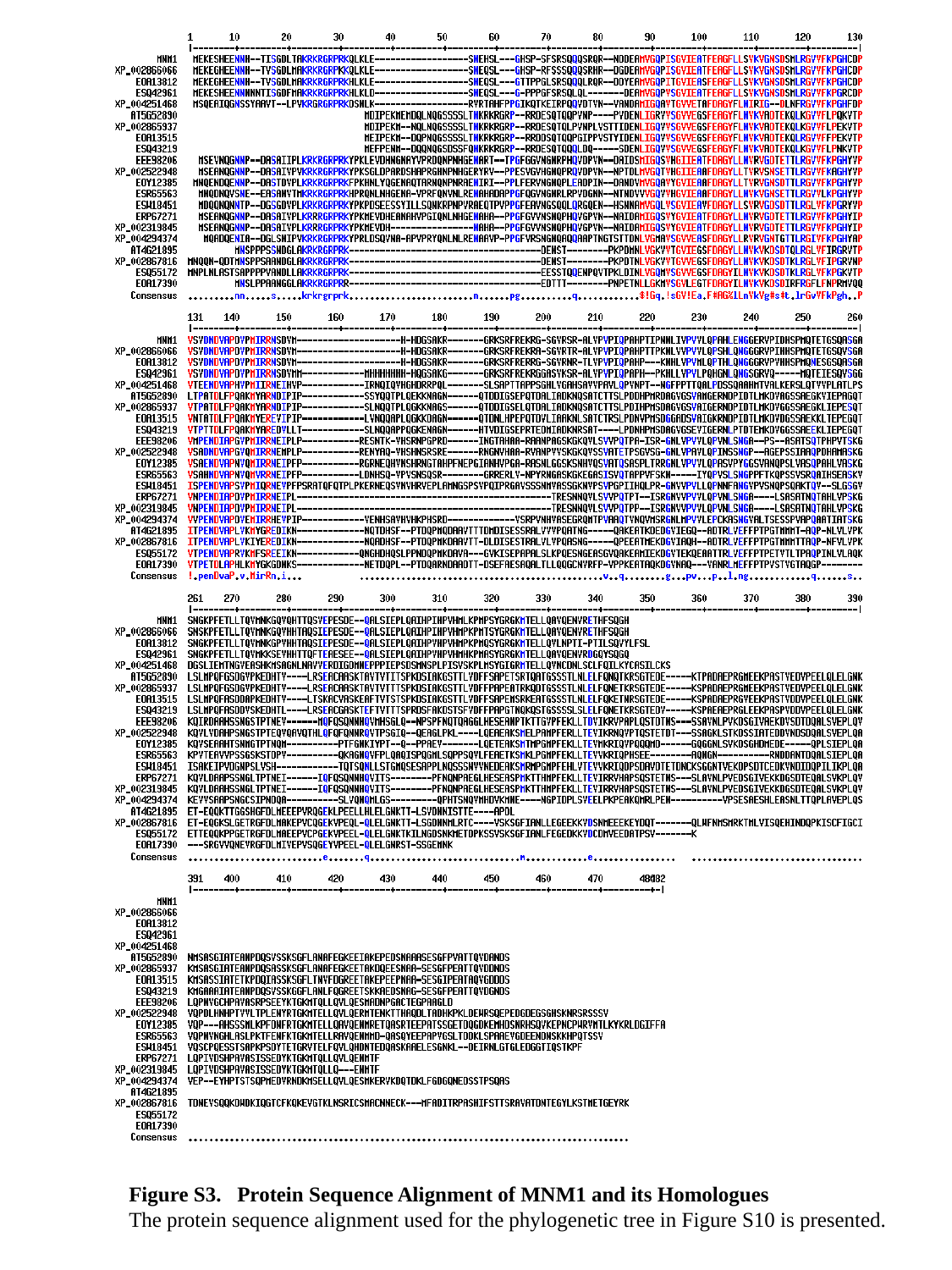

Figure S3. Protein Sequence Alignment of MNM1 and its Homologues
The protein sequence alignment used for the phylogenetic tree in Figure S10 is presented.

## Slide 4
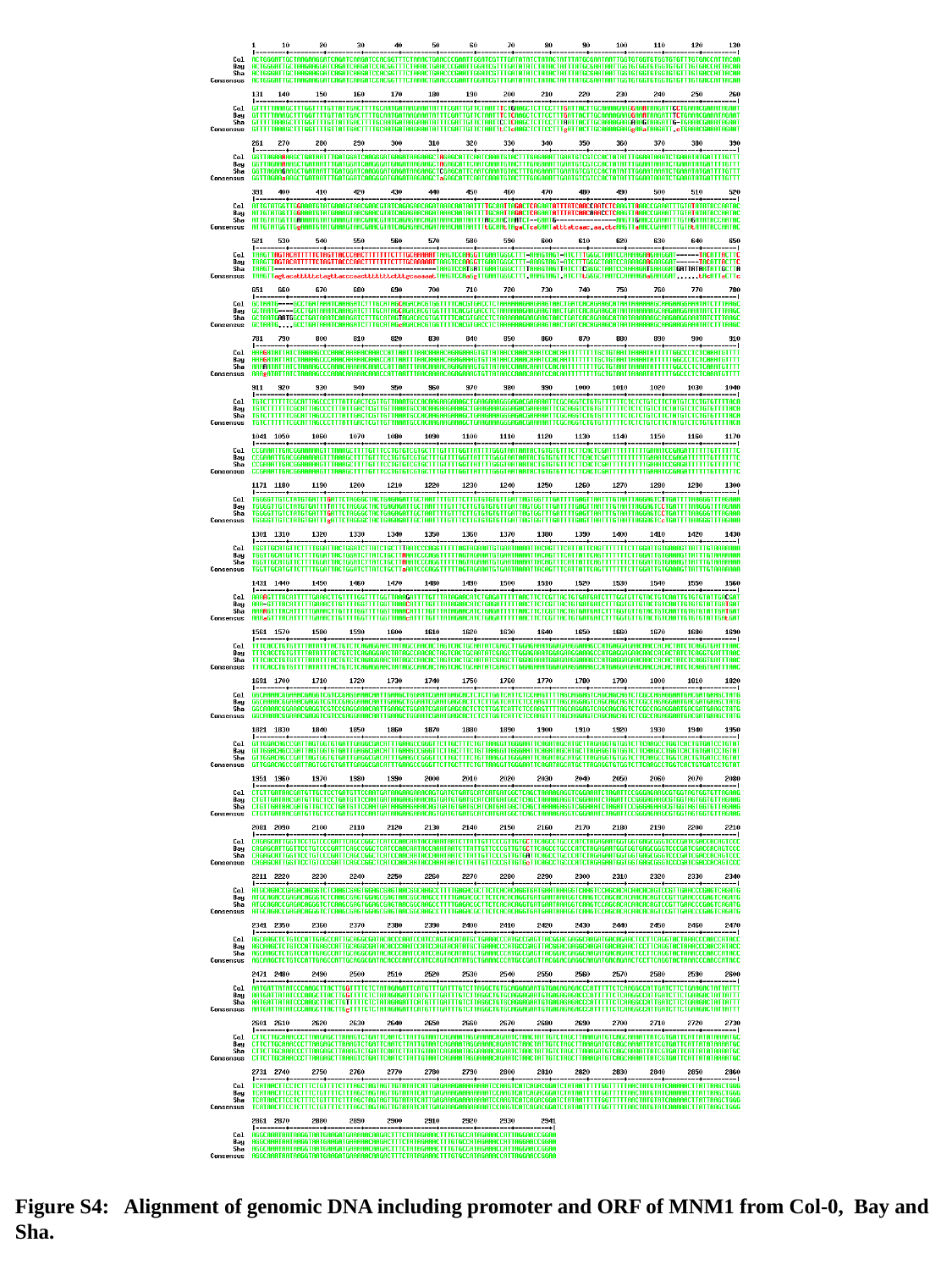

Figure S4: Alignment of genomic DNA including promoter and ORF of MNM1 from Col-0, Bay and Sha.

## Slide 5
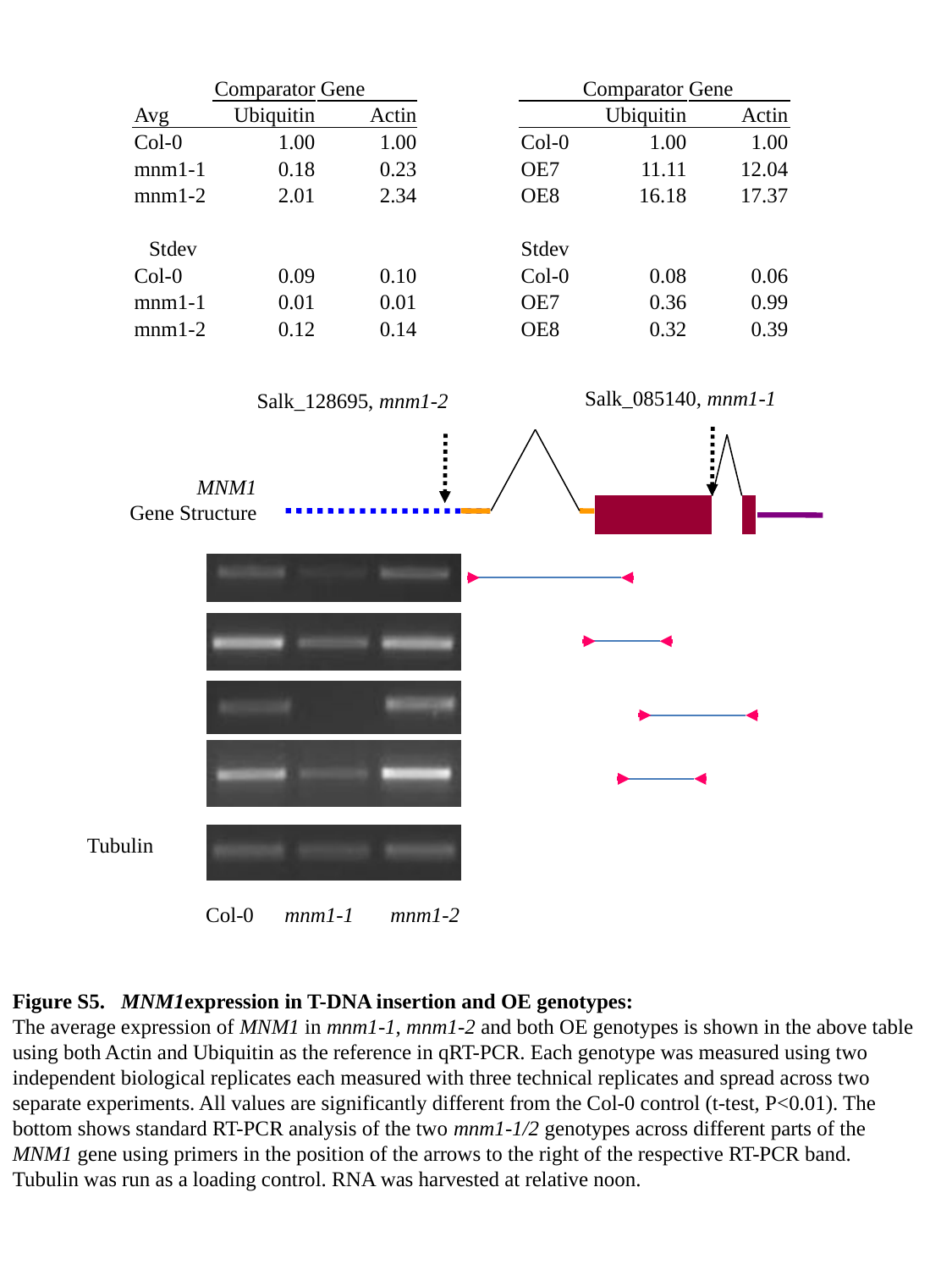

| | Comparator Gene | | | | Comparator Gene | |
| --- | --- | --- | --- | --- | --- | --- |
| Avg | Ubiquitin | Actin | | | Ubiquitin | Actin |
| Col-0 | 1.00 | 1.00 | | Col-0 | 1.00 | 1.00 |
| mnm1-1 | 0.18 | 0.23 | | OE7 | 11.11 | 12.04 |
| mnm1-2 | 2.01 | 2.34 | | OE8 | 16.18 | 17.37 |
| | | | | | | |
| Stdev | | | | Stdev | | |
| Col-0 | 0.09 | 0.10 | | Col-0 | 0.08 | 0.06 |
| mnm1-1 | 0.01 | 0.01 | | OE7 | 0.36 | 0.99 |
| mnm1-2 | 0.12 | 0.14 | | OE8 | 0.32 | 0.39 |
Salk_085140, mnm1-1
Salk_128695, mnm1-2
MNM1
Gene Structure
Tubulin
Col-0
mnm1-1
mnm1-2
Figure S5. MNM1expression in T-DNA insertion and OE genotypes:
The average expression of MNM1 in mnm1-1, mnm1-2 and both OE genotypes is shown in the above table using both Actin and Ubiquitin as the reference in qRT-PCR. Each genotype was measured using two independent biological replicates each measured with three technical replicates and spread across two separate experiments. All values are significantly different from the Col-0 control (t-test, P<0.01). The bottom shows standard RT-PCR analysis of the two mnm1-1/2 genotypes across different parts of the MNM1 gene using primers in the position of the arrows to the right of the respective RT-PCR band. Tubulin was run as a loading control. RNA was harvested at relative noon.

## Slide 6
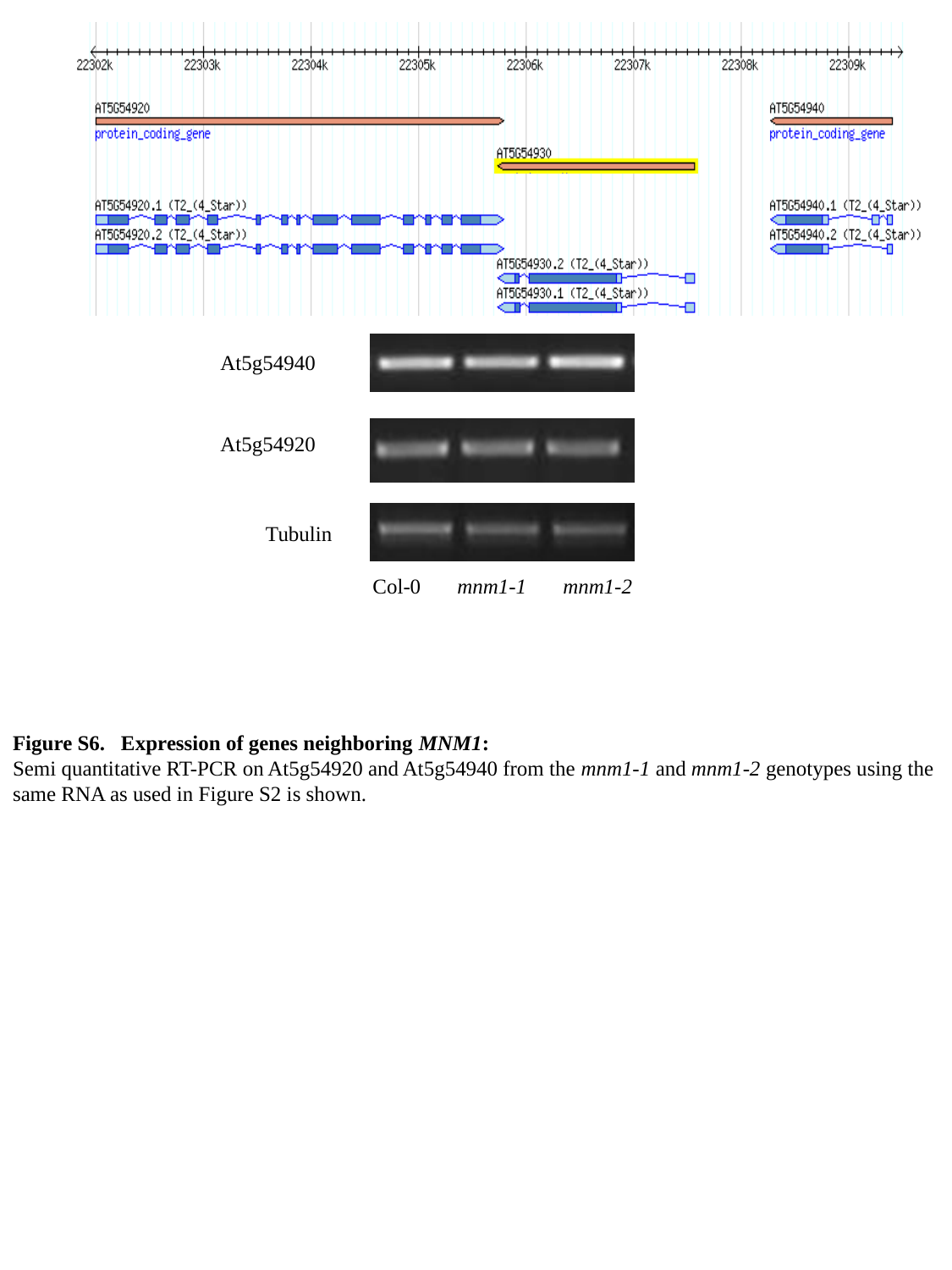

At5g54940
At5g54920
Tubulin
Col-0
mnm1-1
mnm1-2
# Figure S6. Expression of genes neighboring MNM1: Semi quantitative RT-PCR on At5g54920 and At5g54940 from the mnm1-1 and mnm1-2 genotypes using the same RNA as used in Figure S2 is shown.

## Slide 7
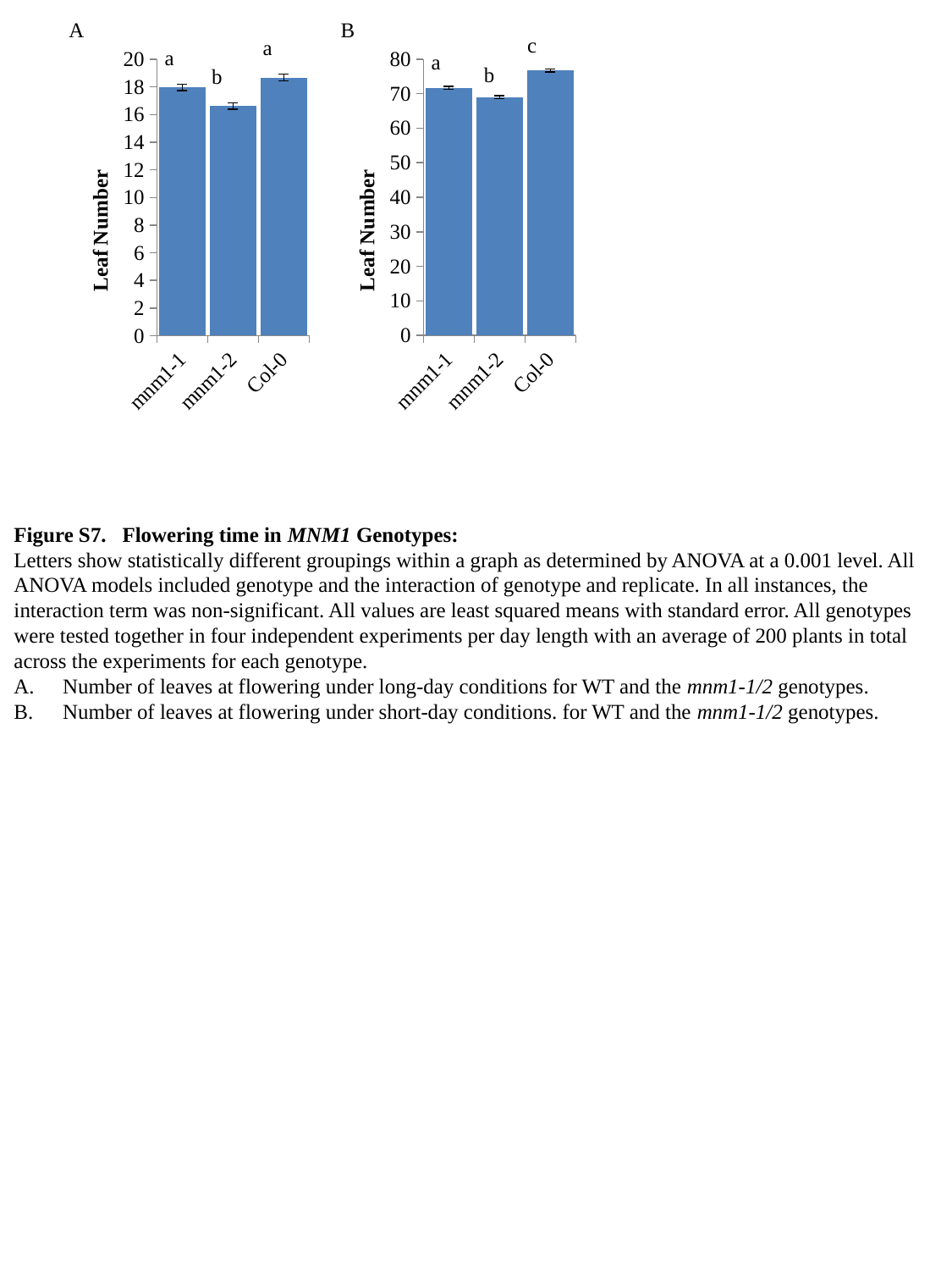

A
B
c
a
### Chart
| Category |
|---|
### Chart
| Category |
|---|a
### Chart
| Category | SD |
|---|---|
| mnm1-1 | 71.6768769 |
| mnm1-2 | 68.9393766 |
| Col-0 | 76.7130281 |
### Chart
| Category | LD |
|---|---|
| mnm1-1 | 17.9644294 |
| mnm1-2 | 16.6115159 |
| Col-0 | 18.6785454 |a
b
b
Figure S7. Flowering time in MNM1 Genotypes:
Letters show statistically different groupings within a graph as determined by ANOVA at a 0.001 level. All ANOVA models included genotype and the interaction of genotype and replicate. In all instances, the interaction term was non-significant. All values are least squared means with standard error. All genotypes were tested together in four independent experiments per day length with an average of 200 plants in total across the experiments for each genotype.
Number of leaves at flowering under long-day conditions for WT and the mnm1-1/2 genotypes.
Number of leaves at flowering under short-day conditions. for WT and the mnm1-1/2 genotypes.

## Slide 8
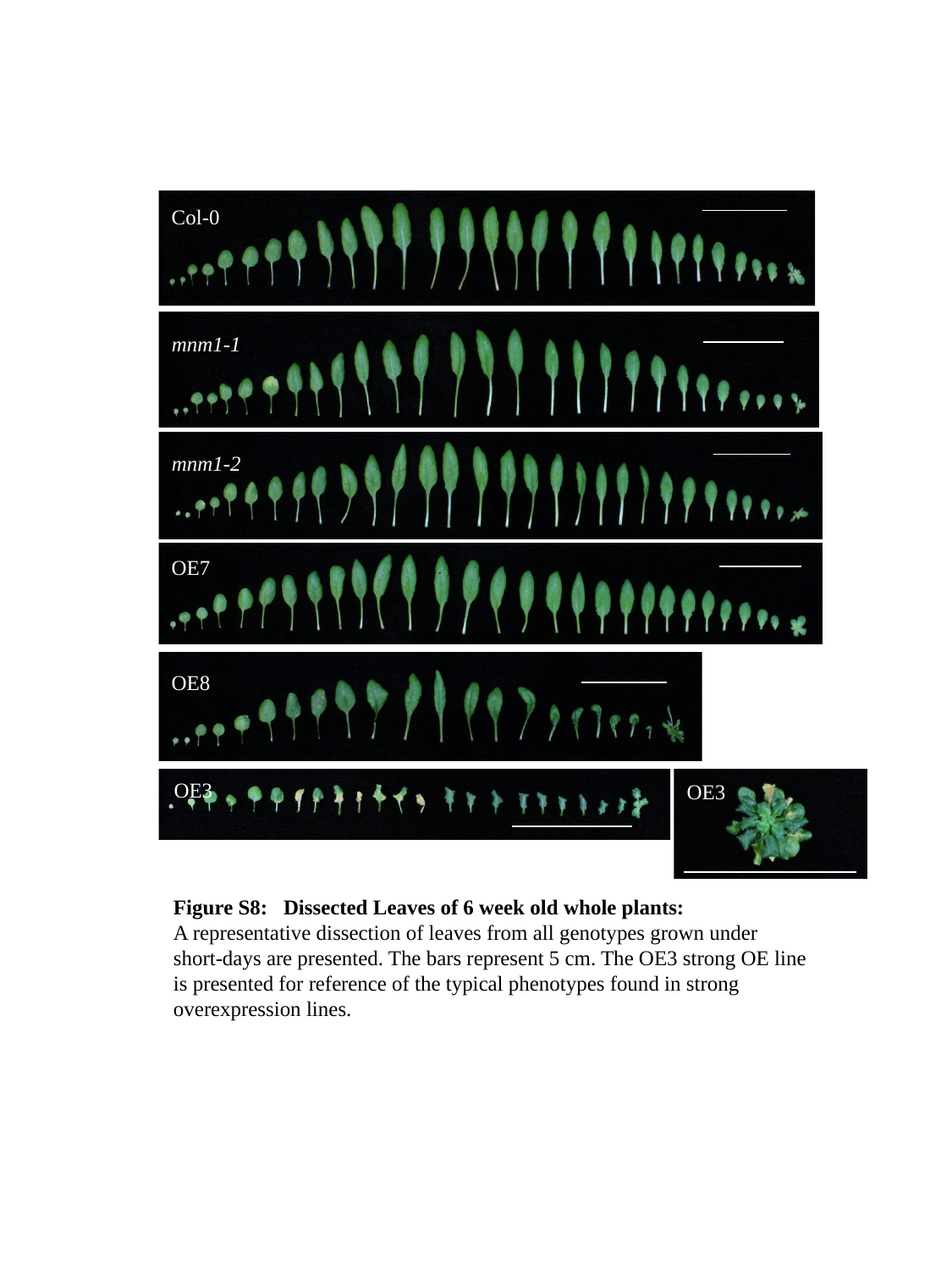

Col-0
mnm1-1
mnm1-2
OE7
OE8
OE3
OE3
Figure S8: Dissected Leaves of 6 week old whole plants:
A representative dissection of leaves from all genotypes grown under short-days are presented. The bars represent 5 cm. The OE3 strong OE line is presented for reference of the typical phenotypes found in strong overexpression lines.

## Slide 9
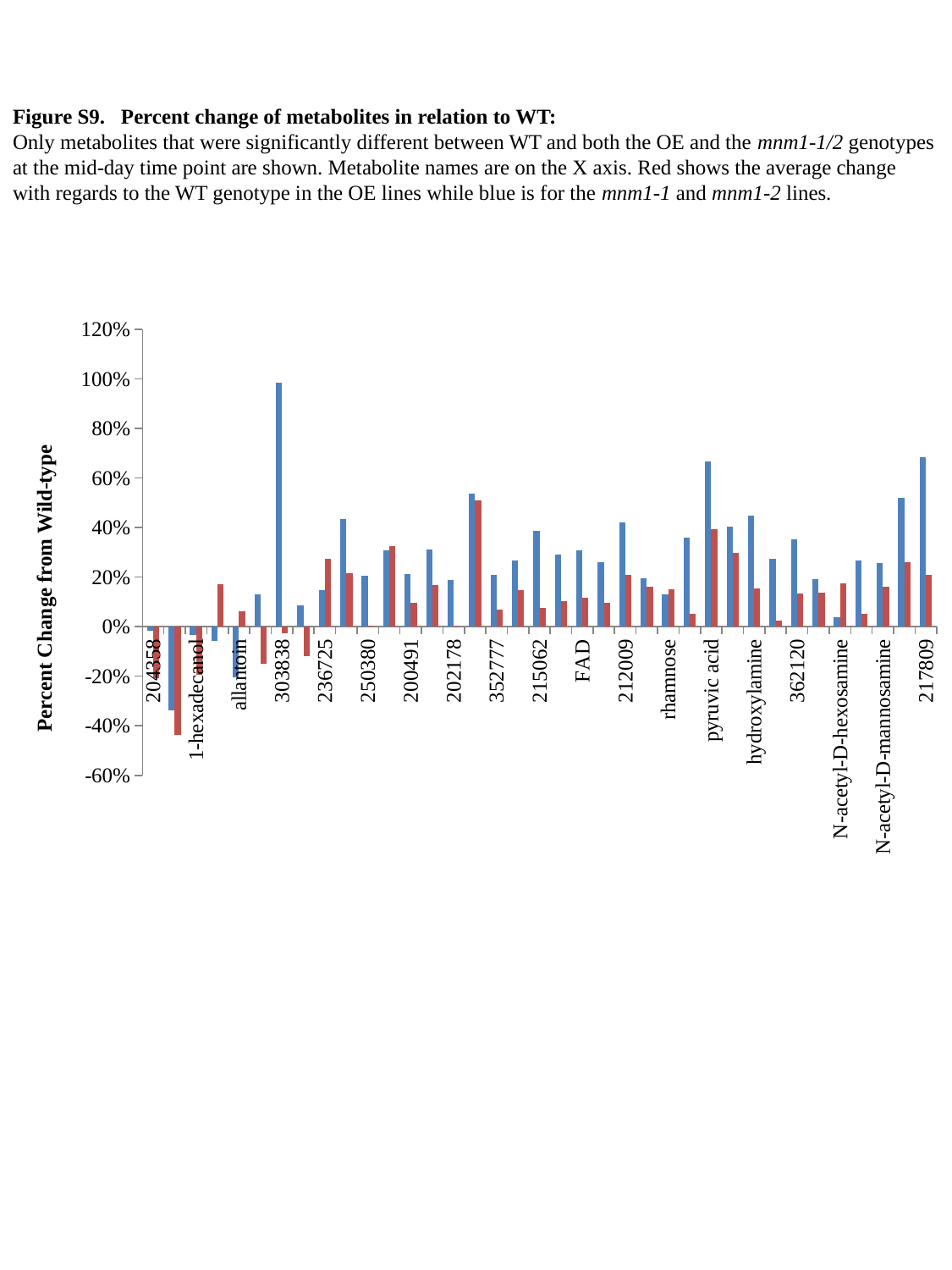

# Figure S9. Percent change of metabolites in relation to WT: Only metabolites that were significantly different between WT and both the OE and the mnm1-1/2 genotypes at the mid-day time point are shown. Metabolite names are on the X axis. Red shows the average change with regards to the WT genotype in the OE lines while blue is for the mnm1-1 and mnm1-2 lines.
### Chart
| Category | KO | OE |
|---|---|---|
| 204358 | -0.01806942463147884 | -0.20851165002377556 |
| 211972 | -0.3371677780235755 | -0.4361944189868217 |
| 1-hexadecanol | -0.03408417308832246 | -0.19462556806955147 |
| succinic acid | -0.05811590931080079 | 0.17025187860805624 |
| allantoin | -0.20439632545931757 | 0.06080489938757655 |
| 280602 | 0.12884784520668427 | -0.1505057167985928 |
| 303838 | 0.9860009332711153 | -0.026189920671955202 |
| 268420 | 0.08539673607203152 | -0.11888013505908836 |
| 236725 | 0.1460901506661686 | 0.2755883451624953 |
| 199239 | 0.4339171974522293 | 0.21528662420382166 |
| 250380 | 0.2042414465281979 | 0.003932244404113733 |
| 225481 | 0.3072828539199098 | 0.32624788494077833 |
| 200491 | 0.21363034911956727 | 0.09718203364692539 |
| 212672 | 0.31297835569088966 | 0.16846350094642415 |
| 202178 | 0.1905196308901442 | 0.000438581102417459 |
| 304346 | 0.538347820944947 | 0.5085743007509905 |
| 352777 | 0.208286969671032 | 0.06883933921081914 |
| 211896 | 0.26738219481340925 | 0.14876660341555978 |
| 215062 | 0.38810855038865405 | 0.07532335385874184 |
| sophorose | 0.29163393558523176 | 0.10428122545168893 |
| FAD | 0.30940115904700577 | 0.11687057308435286 |
| 267647 | 0.2595506182768674 | 0.09517945109078114 |
| 212009 | 0.42022905846317754 | 0.20834998002397123 |
| 199177 | 0.1942075977743085 | 0.16130258996837596 |
| rhamnose | 0.1295223071911114 | 0.15143513596927402 |
| 224586 | 0.35930408472012104 | 0.05219364599092285 |
| pyruvic acid | 0.6684712084347121 | 0.39286293592862936 |
| 437806 | 0.4041526374859708 | 0.2984287317620651 |
| hydroxylamine | 0.4485304195063605 | 0.15504416840059307 |
| 352563 | 0.2750212695561753 | 0.02503308597627263 |
| 362120 | 0.3515840952583457 | 0.13517967254943652 |
| glycero-guloheptose | 0.19153305949383861 | 0.13644494501126275 |
| N-acetyl-D-hexosamine | 0.039758421773910826 | 0.17399687669311917 |
| ferulic acid | 0.2673778389538885 | 0.05196145905024088 |
| N-acetyl-D-mannosamine | 0.2561341084494337 | 0.16067455146837728 |
| enolpyruvate | 0.5186200923787528 | 0.2603926096997691 |
| 217809 | 0.6849587282176705 | 0.20773463772546621 |

## Slide 10
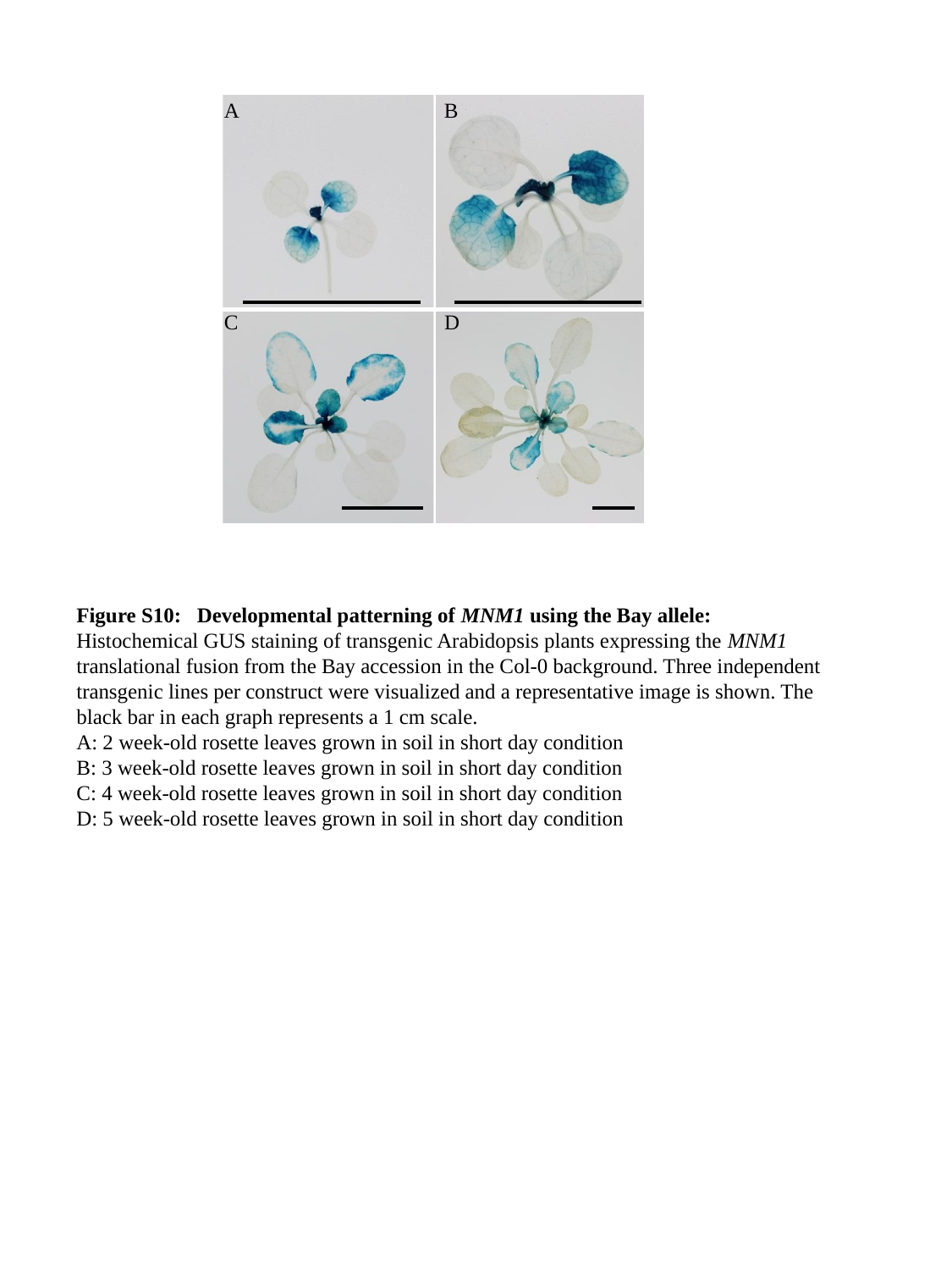

A
B
C
D
Figure S10: Developmental patterning of MNM1 using the Bay allele:
Histochemical GUS staining of transgenic Arabidopsis plants expressing the MNM1 translational fusion from the Bay accession in the Col-0 background. Three independent transgenic lines per construct were visualized and a representative image is shown. The black bar in each graph represents a 1 cm scale.
A: 2 week-old rosette leaves grown in soil in short day condition
B: 3 week-old rosette leaves grown in soil in short day condition
C: 4 week-old rosette leaves grown in soil in short day condition
D: 5 week-old rosette leaves grown in soil in short day condition

## Slide 11
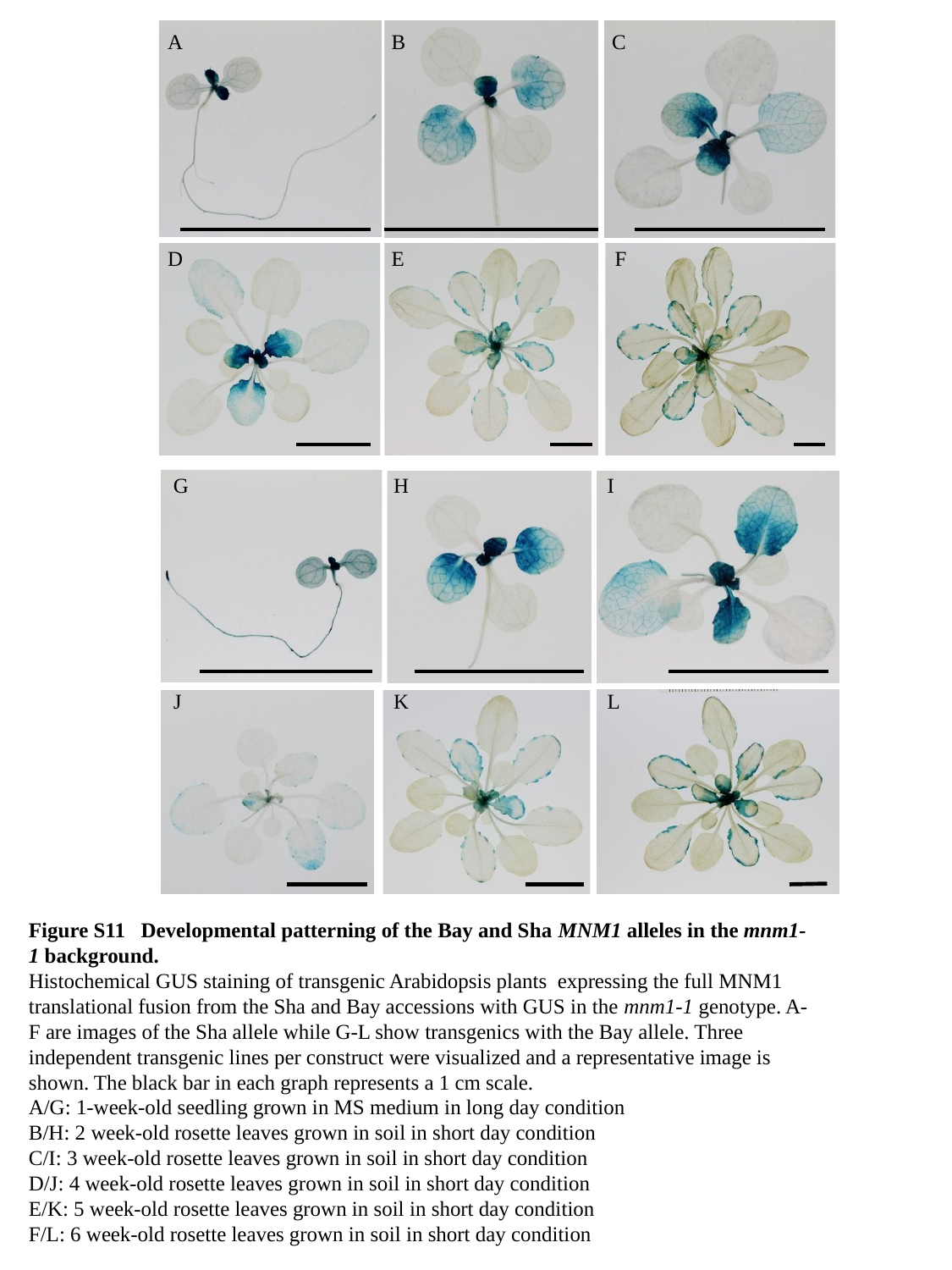

A
B
C
D
E
F
G
H
I
J
K
L
Figure S11 Developmental patterning of the Bay and Sha MNM1 alleles in the mnm1-1 background.
Histochemical GUS staining of transgenic Arabidopsis plants expressing the full MNM1 translational fusion from the Sha and Bay accessions with GUS in the mnm1-1 genotype. A- F are images of the Sha allele while G-L show transgenics with the Bay allele. Three independent transgenic lines per construct were visualized and a representative image is shown. The black bar in each graph represents a 1 cm scale.
A/G: 1-week-old seedling grown in MS medium in long day condition
B/H: 2 week-old rosette leaves grown in soil in short day condition
C/I: 3 week-old rosette leaves grown in soil in short day condition
D/J: 4 week-old rosette leaves grown in soil in short day condition
E/K: 5 week-old rosette leaves grown in soil in short day condition
F/L: 6 week-old rosette leaves grown in soil in short day condition

## Slide 12
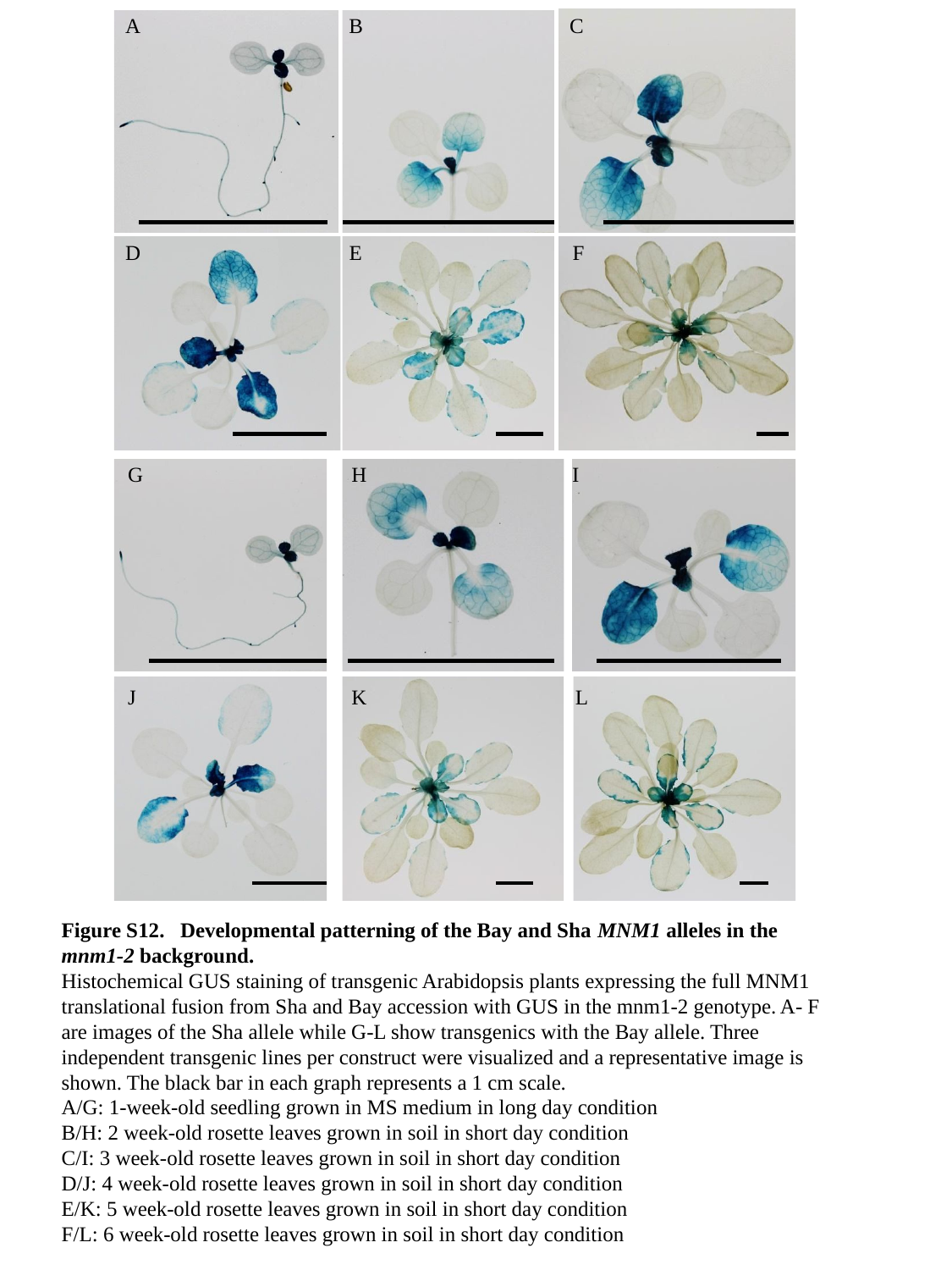

A
B
C
D
E
F
G
H
I
J
K
L
Figure S12. Developmental patterning of the Bay and Sha MNM1 alleles in the mnm1-2 background.
Histochemical GUS staining of transgenic Arabidopsis plants expressing the full MNM1 translational fusion from Sha and Bay accession with GUS in the mnm1-2 genotype. A- F are images of the Sha allele while G-L show transgenics with the Bay allele. Three independent transgenic lines per construct were visualized and a representative image is shown. The black bar in each graph represents a 1 cm scale.
A/G: 1-week-old seedling grown in MS medium in long day condition
B/H: 2 week-old rosette leaves grown in soil in short day condition
C/I: 3 week-old rosette leaves grown in soil in short day condition
D/J: 4 week-old rosette leaves grown in soil in short day condition
E/K: 5 week-old rosette leaves grown in soil in short day condition
F/L: 6 week-old rosette leaves grown in soil in short day condition
